# Recent Advances in AI-Driven Mobile Health Enhancing Healthcare—Narrative Insights into Latest Progress

**DOI:** 10.3390/bioengineering13010054

**Published:** 2025-12-31

**Authors:** Sandra Morelli, Daniele Giansanti

**Affiliations:** Centro IATIS, ISS, via Regina Elena 299, 00161 Rome, Italy; sandra.morelli@iss.it

**Keywords:** mHealth, mobile health apps, AI, artificial intelligence, medical device regulation, AI regulation

## Abstract

Background: The integration of artificial intelligence (AI) into mobile health (mHealth) applications has been accelerated by the widespread adoption of smartphones and recent technological advances, particularly in the wake of the COVID-19 pandemic. This experience has expanded the role of AI-powered apps in real-time health monitoring, early detection, and personalized treatment pathways. Aim: This review aims to summarize recent evidence on the use of AI in healthcare-related mobile applications, with a focus on clinical trends, practical implications, and future directions. Methods: Studies were prioritized based on methodological rigor, with systematic reviews forming the core of the analysis. Additional literature was considered to capture emerging trends and applications where a relevant rigorous screening and scoring procedure was applied to ensure methodological quality and relevance. Only studies addressing healthcare applications, rather than computational or computer science frameworks, were included to reflect the journal’s clinical scope. Results and Discussion: Fifty-six secondary studies were analyzed in detail. Thematic synthesis revealed a post-pandemic shift toward applications targeting mental health, chronic care management, and preventive services. Additional screening showed that, despite their increasing use in clinical contexts, few AI-based apps were formally classified as medical devices. This highlights a gap between technological innovation and regulatory oversight. Ethical concerns—including algorithm transparency, clinical responsibility, and data protection—were frequently reported across studies. Conclusions: This review underscores the growing impact of AI in mobile health, while drawing attention to unresolved challenges related to regulation, safety, and clinical accountability. A more robust integration into health systems will require clearer governance frameworks, validation standards, and interdisciplinary dialogue between developers, clinicians, and regulators.

## 1. Introduction

The introduction is structured to guide the reader from the historical roots of mobile health to the current challenges and opportunities of AI integration. Section 1.1, “Brief story of mHealth”, provides the necessary context by tracing the development of health monitoring from early space programs and wearable sensors to the advent of smartphones, which enabled app-based healthcare. This historical perspective highlights how technological evolution set the stage for AI-driven applications. Section 1.2, “Current State of AI in mHealth”, then focuses on the rapid adoption of AI, particularly accelerated during the COVID-19 pandemic. It reviews applications in telemedicine, chronic disease management, and real-time monitoring, while also identifying critical gaps in evidence, regulatory challenges, and ethical concerns. These observations naturally lead to the key questions that guide the review, highlighting where knowledge is sufficient and where further investigation is needed. Finally, Section 1.3, “Purpose of the narrative review,” explains why synthesizing high-quality secondary studies is timely and necessary. It clarifies the aims of the study, connecting the historical evolution and current state of AI in mHealth to the need for a structured, comprehensive analysis of trends, intervention categories, and implementation challenges.

### 1.1. Brief Story of Mhealth

The integration of artificial intelligence with mobile apps is linked to several key factors. *First*, the concept of mobile health (mHealth) has deep historical roots. *Second*, mobile phones evolved into smartphones with operating systems allowing app installation via platforms like app stores. *Third*, technological advancement and miniaturization enabled increasingly sophisticated features in smaller devices. *Finally*, the COVID-19 pandemic accelerated AI adoption in healthcare, emphasizing the need for innovative tools.

Mobile health began to take shape as early as the 1950s–1960s during space programs involving animals. For example, Laika, the first living creature in space, was monitored with wearable sensors transmitting data to Earth [1,2], analyzed by rudimentary software. As human space missions developed, these systems improved [2,3], laying a foundation for health applications. Before smartphones, physiological monitoring relied on wearable devices, with data analyzed on remote systems using specialized software [4]. The launch of modern smartphones in 2007/2008 [5]—enabling app downloads—marked a turning point. Initially aimed at consumers, apps soon extended to healthcare, transforming health monitoring and management [6].

A 2013 systematic review analyzed 117 studies produced from 2002 to 2012 [6] on mHealth, highlighting key trends: early mHealth relied on basic mobile features like SMS and calls for medication reminders, behavior support, and health education; smartphones’ potential remained largely unexplored; most studies focused on chronic disease management (e.g., diabetes, cardiovascular disease); early research had limited scope with small pilot trials; and studies gradually shifted from feasibility to evaluating real health impacts. This review showed the gradual transition to smartphone-based mHealth, exploring advanced technologies such as apps, sensors, and real-time data collection [6].

A 2019 review on mHealth interventions for First Nations populations examined mental health and suicide prevention [7]. Interventions used SMS and apps to deliver content. While culturally appropriate and acceptable, clinical outcomes were mixed, and evidence for effectiveness remained limited, highlighting the need for further research with stronger designs and larger samples. The COVID-19 pandemic acted as a catalyst for mHealth development. An umbrella review [8], covering publications from January 2020 to April 2022, highlighted the rapid increase in COVID-19 mobile apps, mostly from the USA, UK, and India. Studies were grouped into four clusters: app overview, privacy and security, MARS, and miscellaneous. The review noted gaps in evidence and called for further research on effectiveness and factors contributing to app success.

A scoping review [9] specifically examined mobile health applications released and used between March 2020 and February 2022. It categorized apps into primary prevention (public education and infection prevention) and secondary prevention (symptom monitoring, remote consultations, and data exchange). These mHealth tools proved effective in supporting remote care, patient monitoring, and healthcare coordination. During the pandemic [10], a “gray zone” of apps emerged, where non-medical apps (e.g., fitness or wellness apps) could resemble medical devices without regulation, creating potential risks for patient safety.

### 1.2. Current State of AI in mHealth

The integration of artificial intelligence (AI) in mobile health (mHealth) has accelerated sharply after 2020 in coincidence with the COVID-19 pandemic [11]. The urgent need for rapid, scalable solutions drove AI adoption across multiple areas of healthcare, including patient management, remote diagnosis, and disease monitoring. During the pandemic, AI applications were used to track virus spread, analyze data from mobile apps, wearable devices, and sensors [12], and employ machine learning algorithms to predict case trends, personalize treatment plans, and analyze patient-reported symptoms [13].

AI also enhanced telemedicine, automating diagnostic processes and improving the accuracy of remote assessments. It supported chronic disease management through real-time monitoring and dynamic treatment adjustments [14]. These applications illustrate significant benefits in accessibility, efficiency, and patient engagement, particularly in resource-limited or remote settings.

Despite these advances, important gaps remain in the literature. Many studies focus on technical feasibility or short-term outcomes, leaving long-term clinical effectiveness largely unaddressed. Research is often fragmented across disease areas, and there is limited consensus on the standards for evaluating AI interventions in mHealth. Critical issues regarding ethics, regulation, and patient safety remain inconsistently explored. Concerns include patient autonomy in AI-driven decisions, algorithmic bias, and the risk of dehumanizing care [15,16], as well as data privacy, transparency, and ownership. The phenomenon of the “gray zone” of apps, where non-medical apps can resemble regulated medical devices, further complicates the regulatory landscape and raises potential safety risks. The emerging field of algor-ethics has begun to address these challenges, focusing on the ethical and societal implications of algorithmic decision-making, particularly when AI assists or substitutes for healthcare professionals [17].

The COVID-19 experience also demonstrated that AI-driven mHealth can expand healthcare capacity beyond acute crises. Today, AI supports chronic disease management [18] and telemedicine interventions [19], integrating real-time monitoring, personalized treatment, and improved healthcare workflows. These tools make healthcare more accessible and efficient, especially in remote or underserved areas, and illustrate the transformative potential of AI in modern healthcare systems [12,13,14].

Given these developments and the remaining uncertainties, several key questions naturally emerge from the literature and the observed gaps:

- Current trends: What are the main trends in AI use across different mHealth domains, and how rapidly is the technology being integrated into everyday healthcare practices?
- Categorization and impact: How can AI applications in mHealth be categorized, and which types show the greatest promise for improving patient outcomes and enhancing healthcare efficiency?
- Opportunities and challenges: What are the main opportunities offered by AI, and what challenges—including regulatory, ethical, and implementation-related barriers—need to be addressed to ensure safe, equitable, and effective deployment?

These questions reflect both the progress achieved and the critical gaps in current knowledge. By focusing on trends, categories, and barriers, they provide a clear framework for understanding where AI in mHealth is most effective, where further research is needed, and what factors are crucial for its responsible integration into healthcare.

### 1.3. Purpose of the Narrative Review

Based on the rapid integration of artificial intelligence (AI) in mobile health (mHealth) during and after the COVID-19 pandemic, there is a clear need to take stock of the current state of AI utilization in this field.

This study aims to analyze and synthesize high-quality secondary studies on AI applications in mHealth, providing an overarching view of how AI is being integrated, what trends have emerged, and where knowledge gaps remain.

The specific objectives are to

- Trends: Examine the evolution and current trends in AI use in mHealth, including key areas of application such as chronic disease management, telemedicine, and mental health.
- Categorization: Classify AI-based mobile health tools and interventions, identifying those that are most effective and those that are emerging.
- Opportunities and Challenges: Evaluate the opportunities AI offers for improving healthcare delivery and accessibility, and address key challenges, including data privacy, regulatory frameworks, and healthcare system integration.

By summarizing current evidence, this review provides a comprehensive resource for understanding the landscape of AI in mHealth and offers insights into its potential future directions.

## 2. Methods

### 2.1. Narrative Review Approach, Search Strategy, and Quality Assessment

This narrative review was conducted following the principles of transparency and methodological consistency outlined in the Narrative Review Checklist ANDJ CL and a structured quality control procedure [20]. The aim was to synthesize high-quality evidence on the integration of AI in mHealth applications, with a primary focus on clinical relevance and healthcare impact rather than purely computational aspects.

#### 2.1.1. Search Strategy, Study Selection, and Scope

The literature search targeted studies at the intersection of mHealth technologies and AI, prioritizing applications with tangible clinical or translational value. Searches were conducted in PubMed and Scopus, covering publications in English up to 31 July 2025. Multiple combinations of search terms were employed, organized by specific focus areas as summarized in Table 1. This approach ensured coverage of both general AI trends and domain-specific applications while maintaining focus on real-world healthcare impact.

The review primarily relies on high-quality evidence identified through a structured search strategy. Although database filters for systematic reviews were applied, some retrieved records were not formally systematic reviews, but were included because the system recognized them as following some type of systematic approach. These were nevertheless included when they provided relevant conceptual, clinical, or methodological elements capable of adding value to the study.

It is important to clarify that the objective of this narrative synthesis is not to count primary studies or generate quantitative estimates. Therefore, potential overlap of primary studies across the included reviews was not treated as a limitation. Instead, the focus is on identifying key themes, clinical patterns, and practical insights, emphasizing the differential contribution of systematic versus non-systematic evidence.

By combining well-established topics from secondary studies this approach allows for a comprehensive thematic evaluation and provides a clinically meaningful, forward-looking synthesis of AI applications in mHealth.

The categorization presented in Table 1 represents a deliberate effort to organize the diverse applications of artificial intelligence in mobile health into coherent focus areas. This work is part of a broader institutional research initiative, which extends beyond the scope of this single study and is being pursued across multiple complementary fronts. These efforts aim to map the evolving landscape of AI in mHealth, support strategic decision-making, and provide insights for clinical practice, policy development, and future research directions.

The table distinguishes five key focus areas—General AI in mHealth, Machine Learning applications, Deep Learning & Neural Networks, Telemedicine & Remote Monitoring, and Specific Clinical Domains—each defined by a set of Mobile Health terms and AI-related terms. Some overlap exists between categories, reflecting the interconnected and interdisciplinary nature of AI applications in mHealth. Terms such as “wearable devices” or “telemedicine” are included selectively where contextually relevant, ensuring that both general and specialized applications are captured.

By structuring the literature in this way, the table serves multiple purposes: it provides a clear snapshot of the current research landscape, facilitates comparative analyses between domains, and establishes a reusable framework for future updates and institutional data collection initiatives. In this context, the categorization supports not only the narrative synthesis presented here but also broader ongoing investigations across diverse research fronts, highlighting emerging trends and opportunities in AI-driven mobile health.

#### 2.1.2. Selection and Qualification of Reviews

Once the database of potential studies was assembled, a multi-step selection and quality assessment process was applied. This approach was adapted from established methods previously used for narrative reviews in related fields, and tailored to the clinical and translational focus of AI in mHealth.

*Algorithm 1: Selection and Qualification Process*

*1.*  *Define inclusion criteria*
  ∘ Primarily systematic reviews and meta-analyses were considered to ensure a high level of methodological rigor and robustness of evidence.
  ∘ In addition, a limited number of non-systematic (narrative or scoping) reviews were included to capture emerging applications, innovative trends, and areas where systematic evidence is still limited.
  ∘ Studies had to focus on clinical or healthcare applications of AI in mHealth, excluding purely technical or algorithmic works without tangible clinical outcomes.
  ∘ Detailed inclusion/exclusion criteria are reported in Table 2.
*2.*  *Initial screening*
  ∘ Titles and abstracts were screened independently by two reviewers.
  ∘ Studies irrelevant to clinical mHealth applications, such as purely computational benchmarks or software architecture reports, were excluded.
*3.*  *Evaluation parameters*
  Each study was assessed using six core parameters:
  ∘ N1: Clear rationale—The study clearly explains the background, objectives, and significance.
  ∘ N2: Adequate research design—Methodology is appropriate to answer the research question.
  ∘ N3: Clearly described methodology—Data collection, analysis, and interpretation are transparent and replicable.
  ∘ N4: Well-presented results—Results are clearly described, with tables, figures, or statistical analysis supporting conclusions.
  ∘ N5: Conclusions justified by results—Conclusions are evidence-based and logically derived.
  ∘ N6: Disclosure of conflicts of interest—Conflicts of interest are explicitly reported.
*4.*  *Scoring system*
  ∘ N1–N5 were scored on a 1–5 scale (1 = poor, 5 = excellent).
  ∘ N6 was assessed as Yes/No (Yes = disclosed; No = not disclosed).
*5.*  *Preselection of studies*
  ∘ Only studies with N1–N5 > 3 and N6 = Yes were preselected.
  ∘ This ensured inclusion of methodologically robust studies with transparency in conflicts of interest.
*6.*  *Final synthesis*
  ∘ Preselected studies were included in the narrative synthesis, providing a clinically focused overview of trends, categories, and challenges in AI-driven mHealth.
  ∘ The synthesis emphasized post-pandemic developments, highlighting how technological innovation intersects with practical healthcare applications.

### 2.2. Screening Team and Reliability Assessment

The screening and selection of studies for this narrative review were conducted with a focus on methodological rigor, clinical relevance, and transparency. The process emphasized bioengineering and translational applications of AI in mobile health, rather than purely computational or technical studies.

*Screening Team:*

The review was conducted by two reviewers, the authors of this study (S.M. and D.G.), each with over 25 years of experience in bioengineering, telemedicine, data science, and regulatory aspects of digital medical technologies. The two authors screened the identified papers for titles and abstracts independently and in a blind manner (using the Rayyan tool) and extracted data from the full texts of the selected papers. Disagreements were resolved through discussion and consensus.

*Screening Protocol:*

All records were initially imported into *Rayyan* (https://www.rayyan.ai, access on 14 November 2025), a web-based tool designed for systematic and scoping reviews. *Rayyan* facilitated deduplication, independent screening of titles and abstracts, and the application of inclusion/exclusion criteria. Table 2 reports the screening inclusion/exclusion protocol.

*Quality Assessment Procedure:*

Each study passing the initial screening underwent a quality assessment using the algorithm described in Algorithm 1. The two reviewers independently scored studies across six domains: rationale clarity (N1), research design (N2), methodology (N3), presentation of results (N4), validity of conclusions (N5), and disclosure of conflicts of interest (N6).

- For N1–N5, a 5-point scale was applied: 1 = Poor, 2 = Fair, 3 = Good, 4 = Very Good, 5 = Excellent.
- N6 was assessed binary (Yes/No), depending on whether conflicts of interest were explicitly disclosed.

All full-text studies were independently assessed by two reviewers using the six-parameter scale (N1–N6). Only studies meeting all thresholds for both reviewers were included in the narrative synthesis. Detailed scoring for the included studies is provided in anonymized form in Supplementary Table S1. Additional information on the assessment process is described in the Supplementary Materials.

This structured, yet flexible, approach ensures that the narrative synthesis is based on high-quality, clinically relevant evidence, highlighting both the strengths and limitations of current AI applications in mobile health.

## 3. Results

The *rationale of the results* is organized to offer a comprehensive, clinically oriented overview of how AI and mHealth applications are transforming healthcare. The structure follows a logical progression: from research trends to clinical insights, and finally to thematic categorization and opportunities for further investigation. This organization allows readers to appreciate both the breadth of the field and the specific contributions of AI-driven mHealth technologies across diverse healthcare domains.

*Section 3.1* Study Selection Flow outlines the approach that guided the identification of studies for this narrative review. The process focused on high-quality, clinically relevant review studies in AI-driven mHealth applications, prioritizing studies that provide insights into patient care, clinical decision-making, and healthcare delivery. This approach establishes a solid foundation for presenting results that are directly meaningful and applicable to healthcare practice, rather than centered on technical or algorithmic development.

*Section 3.2* highlights the temporal and quantitative trends in AI-driven mHealth research, illustrating the growing attention to these technologies and their progressive integration into healthcare systems. This perspective provides context for understanding how innovations have evolved and how the evidence base has expanded over time.

*Section 3.3* synthesizes the selected studies, emphasizing common clinical messages and emerging themes. The review identifies how AI and mHealth platforms contribute to disease prevention, early detection, personalized treatment, and real-time patient monitoring. The categorization into macro-areas allows a structured understanding of the diverse applications, spanning mental health, chronic disease management, diagnostics, public health, and medical informatics. By integrating historical and recent insights, this synthesis highlights recurring patterns, areas of strong evidence, and domains where AI and mHealth are already impacting patient care.

*Section 3.4* explores opportunities and areas needing further investigation, highlighting gaps in the current evidence base and potential directions for future research. This forward-looking perspective emphasizes the transformative potential of AI-powered mHealth platforms in enhancing clinical decision-making, expanding access to care, supporting underserved populations, and improving health outcomes.

Overall, the results provide a clinically grounded, evidence-based synthesis, showing the current scope of AI and mHealth applications and guiding interpretation of how these technologies are reshaping healthcare practices and informing future innovation. By combining research trends, thematic insights, and structured categorization, the review offers a comprehensive roadmap for understanding the evolving role of AI in digital health.

### 3.1. Study Selection Flow

Although a structured selection process is not strictly required for narrative syntheses, we nonetheless adopted one to ensure transparency and reproducibility. Specifically, we considered relevant elements extracted from the literature that provided conceptual, clinical, or methodological insights pertinent to AI-based mobile health applications.

We conducted a targeted literature search in PubMed and Scopus, chosen for their broad and complementary coverage of biomedical, bioengineering, and digital health research. The combined search retrieved 661 records in total, including overlapping entries across databases. After removing 103 duplicates, 558 unique records were retained for subsequent screening.

These records underwent a screening focused on clinical relevance, bioengineering applicability, and the substantive value of the extracted elements, rather than on publication type. The aim was to prioritize contributions capable of informing the design, evaluation, and implementation of AI-driven mobile health tools, rather than studies focused solely on algorithmic development or technical optimization. During this phase, 402 records were excluded, resulting in 156 records eligible for full-text evaluation. Discrepancies between the two reviewers were resolved through discussion, and consensus was always reached.

In the full-text assessment, the remaining records were evaluated for quality, recency, and clinical contribution. During the full-text assessment, all 156 records were subjected to the six-parameter quality evaluation (N1–N6). Of these, 100 records did not meet the inclusion thresholds. During this evaluation, it also emerged that 30 had findings largely incorporated into more recent contributions, and 70 were superseded by newer elements providing more comprehensive, clinically and translationally oriented evidence.

Ultimately, 56 key elements [21,22,23,24,25,26,27,28,29,30,31,32,33,34,35,36,37,38,39,40,41,42,43,44,45,46,47,48,49,50,51,52,53,54,55,56,57,58,59,60,61,62,63,64,65,66,67,68,69,70,71,72,73,74,75,76] were included in the narrative synthesis. These elements provide both breadth and depth, encompassing AI-driven mobile health applications relevant to clinical practice, patient management, evaluation metrics, and evidence-based interventions. By integrating historical and recent evidence, this synthesis highlights emerging trends, consolidates findings across healthcare settings, and informs future research, clinical decision-making, and digital health policy.

### 3.2. Research Trends in Mobile Apps and Their Use of Artificial Intelligence

To provide a contextual overview of research trends in AI-driven mobile health applications, we conducted a broad search in the PubMed database, focusing exclusively on biomedical literature. This search illustrates the overall growth of publications in the field, highlighting the increasing contribution of AI-enabled interventions over time. These trends serve to contextualize the focused analysis of the 56 studies selected through targeted inclusion criteria reported in Section 2, which ensure relevance, methodological rigor, and clinical applicability.

**Figure 1:**
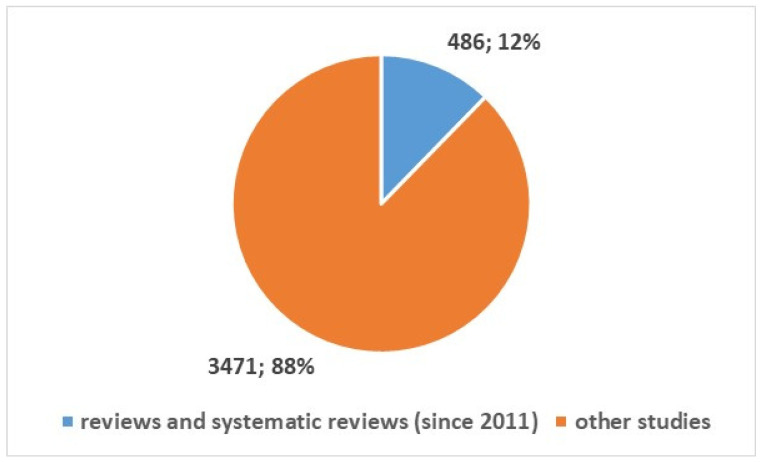
Types of studies found on mobile health apps including AI, since 2002. The number of all studies is 3957, with 12% of reviews and systematic reviews articles published since 2011.

**Figure 2:**
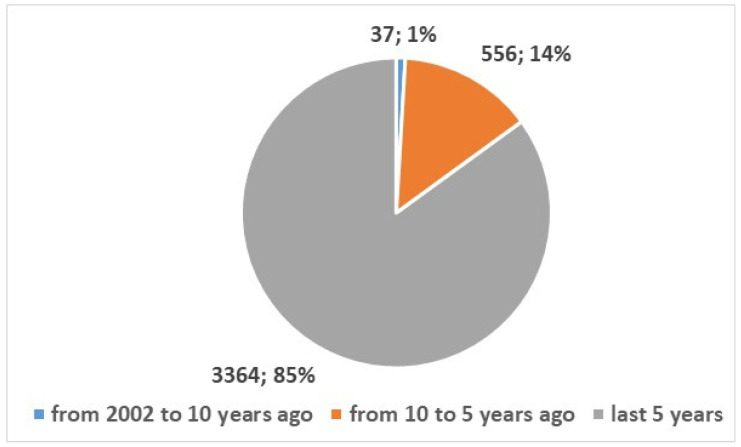
Numerical time trend of all studies published on the use of AI in mobile health apps, since 2002 (number of all studies 3957). The figure shows that the production of such studies was very modest (only 1%) from 2002 to ten years ago, then increased significantly over the last ten years (14% of studies published between 10 and 5 years ago), and has grown impressively in the last five years (85%).

**Figure 3:**
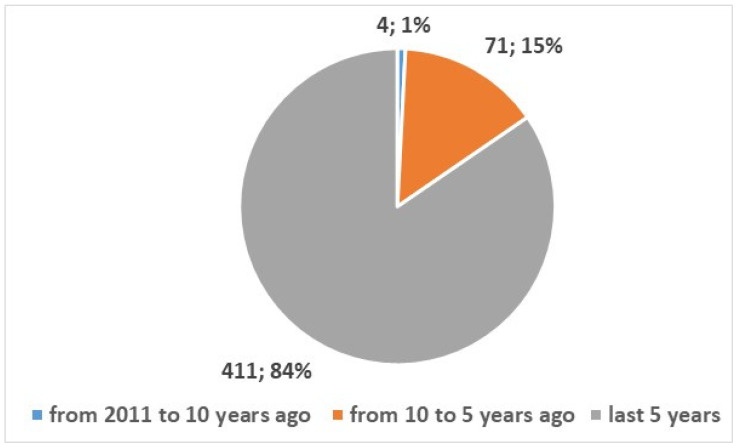
Numerical time trend of review studies on the use of AI in mobile health apps, since 2011 (number of all reviews 486). It should be noted that the number of such studies was very irrelevant (only 1%) from 2011 to ten years ago, then increased meaningfully over the last ten years (15% of studies published between 10 and 5 years ago), and has increased extraordinarily in the last five years (84%).

The search was conducted in the PubMed using the search composite keys in Table S2 (in the Supplementary Materials), position 1, resulting in a total of 3957 studies published since 2002. Figure 1 shows the types of studies: 486 reviews and systematic reviews articles published since 2011 (12%), and 3471 studies of other types. Figure 2 and Figure 3 depict the numerical temporal trend of all studies and review studies respectively.

**Figure 4:**
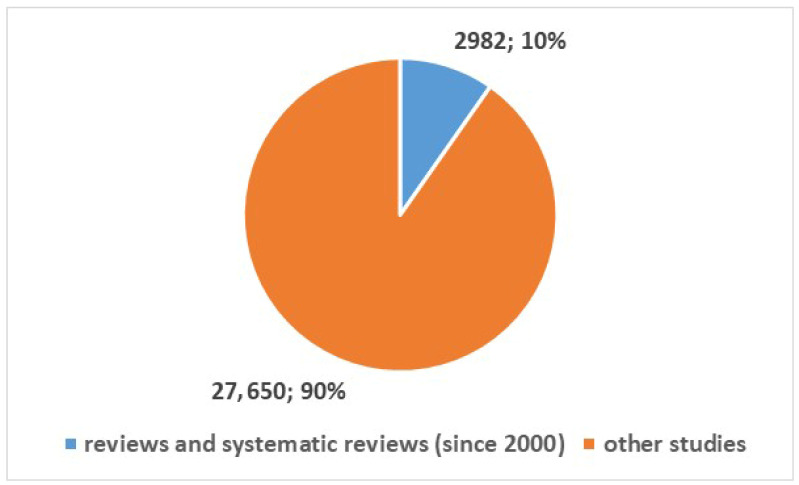
Types of studies published on mobile health apps, since 2000 (number of all studies 30,632).

**Figure 5:**
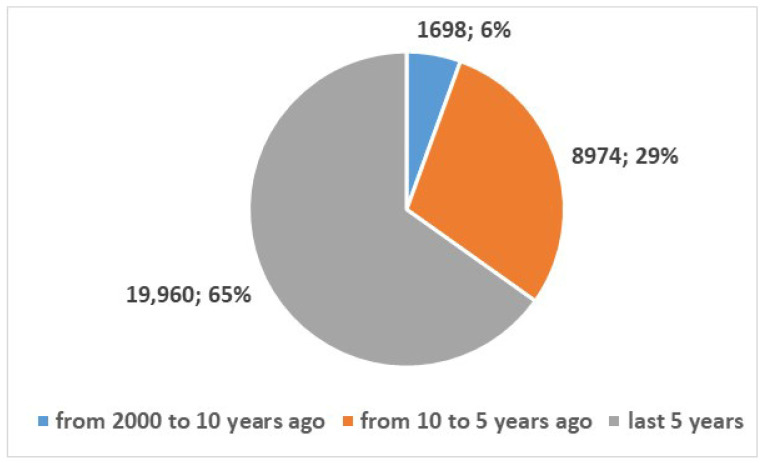
Numerical time trend of all studies published on mobile health apps, since 2000 (number of all studies 30,632). The trend shows that, from 2000 to ten years ago, very few studies were conducted (only 6%). Then, between 10 and 5 years ago, this figure increased to 29%. Finally, in the last five years, the number of studies has become very considerable (65%).

**Figure 6:**
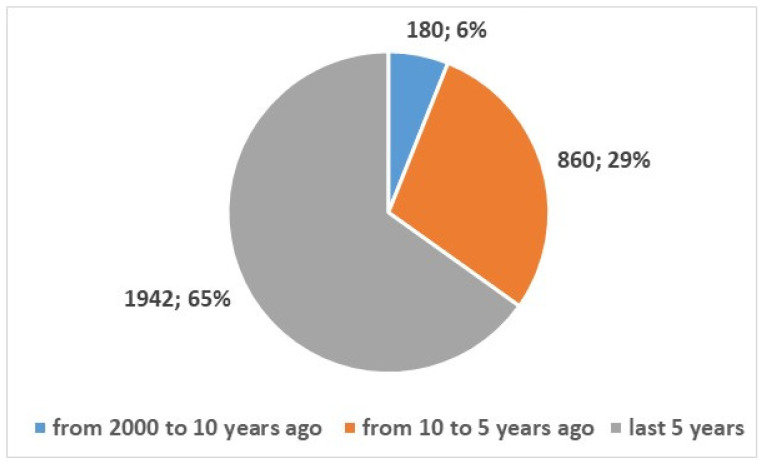
Numerical time trend of review studies on mobile health apps, since 2000 (number of all reviews 2982). As you can see, the number of these studies was negligible (only 6%) between 2000 and ten years ago, then increased over the last ten years (29% of studies published between 10 and 5 years ago), and has risen further in the last five years (65%).

We also wanted to examine the development of health apps for smartphones, so we searched for published studies on this topic using the search term in Table S2, Position 2. This search yielded 30,632 results from 2000 onwards. Of these, 2982 were review articles or systematic reviews (10%), while the remaining 27,650 (90%) were articles from other types of studies (see Figure 4). Figure 5 and Figure 6 represent the numerical temporal trend of all studies and review studies respectively.

**Figure 7:**
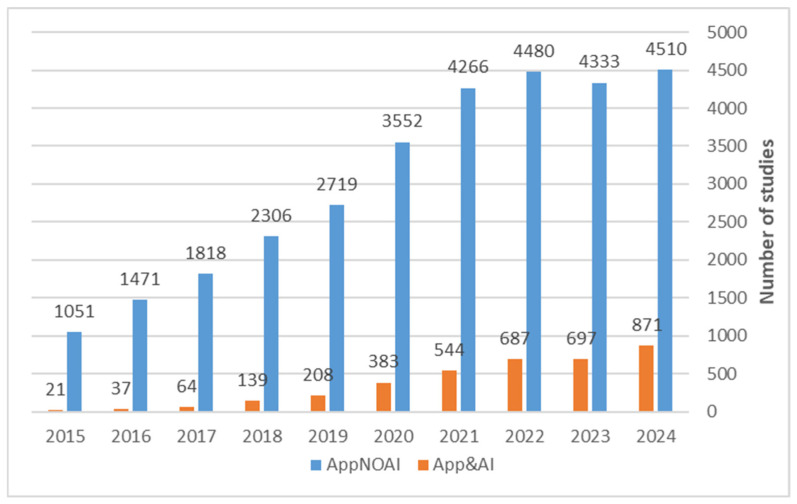
Temporal trend of studies on mobile health apps, without (AppNOAI) and with (APP&AI) AI in the last 10 years (from 2015 to 2024).

Finally, we compared the temporal trend of studies on apps only with those on apps with AI over the last decade, from 2015 to 2024 (Figure 7). The number of both types of studies has been increasing year by year, but the number of studies on apps with AI has increased more rapidly (mean increment per year of 45%) than the number of studies on apps without AI (mean increment per year of 16%). This difference clearly shows that AI-based mobile health apps are developing rapidly thanks to the massive development of this technology.

It must be remarked that while the temporal trends depicted in Figure 1, Figure 2, Figure 3, Figure 4, Figure 5, Figure 6 and Figure 7 reflect the general expansion of AI-related mobile health research, the subsequent analysis concentrates on the 56 studies identified through predefined selection criteria. By focusing on this curated dataset, we provide a detailed and comparable assessment of current applications, methodological approaches, and emerging gaps, complementing the broader bibliographic overview.

### 3.3. Output from the Overview: Common Message, Themes and Categorization

Fifty-six studies were selected using the proposed methodology.

**Figure 8:**
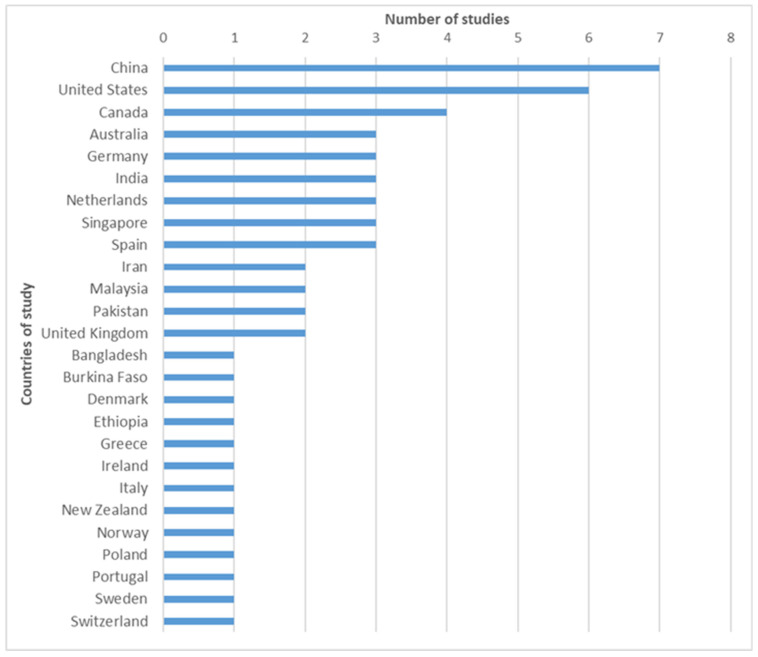
Number of selected studies by country (56 studies from 26 counties).

When we analyzed the countries of authors of selected studies (Figure 8), we found that China, United States and Canada produced the largest number of studies (seven, six, and four respectively), while all other countries produced a maximum of three publications during the analyzed period. For studies conducted by authors from different countries, we have considered the country of the corresponding author.

Table 3 provides an outline of the themes, the contributions of mHealth and AI, the focus, and a brief description of the study. Table 4 presents the emerging categorization into macro-areas.

*Common message*

A clear and recurrent clinical message emerges from the literature on mHealth platforms and artificial intelligence (AI) in healthcare: these technologies are reshaping clinical practice by enhancing disease prevention, diagnostic accuracy, personalized treatment, and real-time patient monitoring.

AI-driven tools enable precise, real-time tracking in mental health, supporting early interventions in vulnerable populations such as youth [21]. In neurology, smartphone-based assessments provide objective evaluations for Parkinson’s disease [26], while digital phenotyping facilitates personalized stress and anxiety management [31].

In disease prevention and early detection, AI applications are proving transformative. Examples include smartphone-assisted oral health screening [25], mobile apps for managing neglected tropical diseases [22], and deep learning algorithms for early oral cancer detection [23]. Telehealth and remote monitoring extend care beyond traditional settings, bridging accessibility gaps, while AI-driven personalized care initiatives face commercialization challenges [53]. Digital health apps support orthopedic diagnoses [56] and ICT-based solutions facilitate COVID-19 remote management [44], illustrating AI-IoT synergy in pandemic response.

Lifestyle and chronic disease management also benefit from AI-enabled interventions, including nutrition support [34] and type 2 diabetes prevention programs [35]. Workplace health promotion leverages AI to target behavioral risks [28], while sensitive health issues are addressed through domestic violence prevention apps [52], fall detection for seniors [55], and surgical site infection prevention tools [54].

Diagnostic and therapeutic innovations are reaching clinical maturity. AI-powered smartphone systems achieve high accuracy in diabetic retinopathy detection [43], tools for obstructive sleep apnea diagnosis are emerging [47], and markerless motion capture enhances rehabilitation [51]. AI-integrated pain management apps optimize patient experience and outcomes [46].

AI also supports knowledge translation and research efficiency, with frameworks for evaluating digital health apps [32], generative AI for systematic reviews [33], and AI-crafted plain language summaries [37], improving dissemination to broader audiences. Recent evidence demonstrates the growing clinical integration of AI-mHealth solutions, including improved cardiovascular risk prediction [60], perioperative support [70], and digitally structured oncology and chronic care pathways [66,72].

Collectively, these studies convey a powerful clinical message: AI-powered mHealth technologies are now essential drivers of a predictive, personalized, participatory, and transformative healthcare paradigm. Their integration promises to revolutionize prevention, diagnosis, treatment, and patient empowerment, advancing equitable and effective care across diverse populations.

*Emerging themes*

The studies summarized in Table 3 demonstrate the wide-ranging contributions of AI and mHealth platforms across multiple healthcare domains. They encompass predictive AI for mental health, smartphone-based tools for chronic disease management, diagnostic aids, and preventive interventions, all aimed at improving outcomes, enhancing disease prevention, and enabling real-time monitoring.

For mental health, predictive AI supports early interventions in youth [21], while digital phenotyping and wearable devices allow continuous monitoring of stress, anxiety, and depression [31]. In neurology, smartphone-based assessments provide objective evaluations for Parkinson’s disease [26], and markerless motion capture enhances rehabilitation [51]. Chronic disease management benefits from personalized AI interventions, including nutrition support [34], type 2 diabetes prevention [35], home-based care for chronic low back pain and COPD [58,59,60,61,62,63,64], and supportive apps for inflammatory bowel disease and spinal cord injury [74,75].

In preventive care, AI-enabled mobile tools improve oral health screening [25], early oral cancer detection [23], diabetic retinopathy [43], and sleep apnea diagnosis [47]. Workplace health initiatives leverage AI to reduce behavioral and mental health risks [28], while domestic violence prevention apps integrate AI for safety and monitoring [52]. Telehealth and ICT-based solutions, including COVID-19 remote management [44] and orthopedic diagnosis [56], highlight the extension of care beyond traditional clinical settings.

Several studies illustrate AI’s role in knowledge translation and system efficiency, such as frameworks for evaluating digital health apps [32], generative AI to streamline systematic reviews [33], and AI-generated plain language summaries [37]. Mobile and AI tools also support public health, addressing gender-based violence [57], promoting vaccination and preventive interventions, and enhancing access in low-resource settings [60,62].

The literature highlights an emerging convergence of AI and mHealth, moving from isolated applications to integrated, adaptive systems capable of predictive, personalized, and participatory care. Across diagnostic, therapeutic, preventive, and rehabilitative domains, these tools are reshaping clinical practice, improving outcomes, and addressing health inequities. Ethical considerations, usability, and contextual fit are increasingly recognized as critical dimensions in the deployment of AI-driven digital health solutions [65,66,67,68,69,70,71,72,73,74,75,76].

Taken together, these studies reinforce the transformative potential of AI-powered mHealth platforms in supporting patient-centered, evidence-based, and accessible healthcare across diverse populations and clinical domains.

*Categorization*

Table 4 is structured to provide a comprehensive overview of the main macro-areas arisen in AI-driven mHealth research. Each row represents a distinct macro-area, reflecting the dominant healthcare domain addressed in the included studies. Studies were grouped based on their primary focus or application, so that, for example, research primarily on mental health falls under “Mental Health & Well-being,” whereas studies on chronic disease management are categorized under “Chronic Diseases & Diagnostics.”

We acknowledge that some conceptual overlap exists—for instance, between “Disease Prevention & Management” and “Public Health,” or across interventions addressing domestic versus workplace violence. This overlap is intentional and reflects the inherently multi-dimensional nature of AI-driven mHealth applications, where a single study may contribute to multiple healthcare objectives. Rather than imposing artificially exclusive boundaries, the categorization prioritizes the dominant theme or clinical focus of each study. The classification is designed to highlight dominant application areas without enforcing strict exclusivity, enable thematic synthesis to identify key patterns and gaps, and provide a structured yet flexible framework that accommodates both established and emerging research areas.

The columns are organized as follows:

- Macro Area: The broad healthcare category where AI and mHealth applications have been applied.
- Number of Included Studies: Total studies contributing to each macro-area, allowing a quick assessment of research volume.
- Evidence Maturity Level: Qualitative assessment of research maturity (e.g., emerging, developing, mature) based on the number and quality of studies.
- Typical Interventions/Focus: Provides a brief summary (1–2 sentences) of the typical interventions, aims, or contributions of the studies in that macro-area.
- Notes/Research Gaps: Highlights areas with limited evidence or underexplored topics, guiding directions for future investigation.
- Key Studies [ ]: Lists the included studies for each macro-area using numerical references corresponding to the reference list.

This organization enables readers to quickly understand both the breadth of research across healthcare domains and the dominant application focus in each area. By integrating study counts, maturity levels, and research gaps as core components, the table facilitates identification of patterns, trends, and priority areas for future AI-mHealth research.

*Key macro-areas include:*

- Mental Health & Well-being: AI and mobile platforms for monitoring and improving mental health, including stress, anxiety, depression, behavioral addictions, suicide risk prediction, and personalized interventions [21,31,41,48,61,63,64,65,66,67,68,69,70].
- Disease Prevention & Management: Digital tools for preventing, diagnosing, and managing diseases such as type 2 diabetes, neglected tropical diseases, domestic violence, Alzheimer’s disease, and physical rehabilitation [22,35,40,50,52].
- AI & Mobile Health Technologies: Methodological frameworks for AI integration in mHealth, chatbots, and diagnostic tools for infectious diseases [32,33,36,37,38,39].
- Cancer & Oral Health: Early detection and self-monitoring of oral health conditions, including oral cancer and dental caries, often leveraging smartphone-based imaging [23,25,27,45,49].
- Chronic Diseases & Diagnostics: AI-supported diagnosis and management of chronic diseases, including diabetes, COPD, cardiovascular conditions, sleep apnea, falls, and pediatric eye screening [30,43,47,54,55,58,60,64,72,74,76].
- Health Data & Outcome Measurement: Patient-generated data for real-world outcome measurement, cognitive assistance, and caregiver support [38,42,50,71].
- Workplace Health: AI-based interventions to promote employee health and prevent disease [28].
- Health Technologies & Innovations: Innovative tools for rehabilitation, forecasting patient arrivals, pain management, and spinal cord injury support [24,46,51,59,75].
- Healthcare Technology Integration: Digital solutions for remote diagnostics, telehealth, ICT-based monitoring, and AI-driven translation in clinical settings [44,53,56,73].
- Technology for Medication Adherence: mHealth apps improving adherence to treatments, particularly in oncology [29].
- Nutrition & Health: Mobile apps supporting nutrition management and healthier behaviors [34].
- Public Health: Smartphone-based interventions addressing public health challenges, such as domestic violence and universal health coverage in low-income settings [52,57,62].
- Surgical Care & Infections: Data-driven technologies preventing surgical site infections [54].
- Medical Informatics: Telehealth startups enhancing healthcare delivery and access [53].

Early research [21,22,23,24,25,26,27,28,29,30,31,32,33,34,35,36,37,38,39,40,41,42,43,44,45,46,47,48,49,50,51,52,53,54,55,56] covered multiple domains including chronic disease management, cancer detection, rehabilitation, telehealth, and initial AI-based mental health tools. More recent studies ([57,58,59,60,61,62,63,64,65,66,67,68,69,70,71,72,73,74,75,76], 2024–2025) show a shift toward mental health, emphasizing AI-powered personalized interventions, real-time monitoring, and integration with chronic disease management.

Overall, the table demonstrates the evolution of AI-driven mHealth research from a broadly distributed landscape to a more focused attention on mental health and well-being, while maintaining substantial contributions in chronic disease care, public health, and innovative technologies. By presenting this structured categorization, Table 4 clarifies the breadth of research, highlights emerging trends, and identifies areas for future investigation.

### 3.4. Opportunities and Areas Needing Broader Investigation

The integration of Artificial Intelligence (AI) and mobile health (mHealth) technologies represents a rapidly evolving frontier in healthcare, offering significant opportunities to enhance disease management, improve patient outcomes, and streamline healthcare delivery. These innovations span predictive tools for mental health, real-time monitoring of chronic conditions, and early detection of infectious diseases. AI-powered mHealth platforms, in particular, have the potential to transform healthcare access and delivery, especially for underserved and remote populations, by identifying risks and patterns in real-time and enabling personalized, timely interventions that can reduce costs and improve outcomes.

For instance, AI can support the prediction and management of mental health conditions such as anxiety and depression in young populations, with mobile apps enabling immediate interventions to promote well-being [21]. In chronic disease management, AI-powered tools enhance monitoring and control of conditions like diabetes, hypertension, and cardiovascular diseases, providing individuals with actionable insights and personalized recommendations [24,28]. Furthermore, AI has a key role in expanding healthcare access in low-resource settings, such as for neglected tropical diseases, which are often underdiagnosed in resource-limited regions [22].

Despite these advantages, integrating AI and mHealth into routine care faces significant challenges. Data privacy and security are critical, particularly when handling sensitive health information [31]. Resistance from healthcare providers, concerns about reliability, integration with existing systems, and implementation costs further limit adoption [25,29]. Accessibility remains a concern in rural and marginalized communities with limited infrastructure.

Ethical and regulatory issues are also central. Clear frameworks are needed to ensure AI tools are effective, equitable, and transparent, addressing potential biases and establishing governance for their use in healthcare [33]. Achieving regulatory approval for AI-driven technologies, particularly in mental health and chronic disease management, remains a complex and ongoing challenge.

Recent studies from late 2024 through 2025 [57,58,59,60,61,62,63,64,65,66,67,68,69,70,71,72,73,74,75,76] highlight the rapid growth of research in this area, illustrating how AI and mHealth are reshaping healthcare delivery across clinical and public health domains. AI applications have improved diagnostic accuracy for pediatric ocular diseases [60] and obstructive sleep apnea [76], and mobile apps for chronic disease self-management, including type 2 diabetes [58] and COPD [64], enable home-based monitoring and personalized interventions. Wearable biosensors combined with AI support real-time mental health monitoring [66], and AI-enhanced ecological momentary assessment offers promising tools for suicide prevention [67].

However, challenges persist. Data privacy, equitable access, algorithmic bias, and the digital divide remain key barriers [62,69,72]. Rigorous validation and standardization are essential to ensure clinical reliability and utility [61,70].

Overall, these studies depict a dynamic digital health landscape where AI and mHealth technologies offer transformative potential for patient-centered care. Table 5 summarizes the opportunities and challenges identified, highlighting areas for impactful implementation and barriers that must be addressed for safe, ethical, and effective adoption.

## 4. Discussion

*Logical progression and integration of the discussion*

To provide a coherent and comprehensive analysis, the discussion follows a logical progression that both complements and extends the results presented in Section 3. The findings derived from the overview of secondary studies inform evidence-based insights, highlight recurring clinical and technological themes, and identify gaps and areas for improvement in AI-powered mHealth applications. The discussion is structured to address multiple interconnected dimensions, including *clinical added value, comparison with prior literature, real-world impact on practice and policy, regulatory and governance issues, quality and usability aspects, technical standards and norms, regulatory gray zones, study limitations, and future research priorities*. This structure ensures a clear link between the results and their practical, clinical, technical, and regulatory implications. In this way, the discussion not only synthesizes the evidence but also integrates it with standards, norms, and best practices, offering a holistic perspective to guide future research, implementation, and governance of AI-driven digital health tools.

*Organization of the discussion*

The discussion is organized into the following sections to provide a comprehensive analysis of the findings and their implications within the field:

*Section 4.1* “*Summary of key findings*” presents the main insights from the included studies, highlighting AI’s transformative role in mHealth across disease prevention, diagnosis, and management. It emphasizes emerging categorizations, thematic trends, and the balance between opportunities and challenges, including clinical impact, personalization, and accessibility improvements.

*Section 4.2* “*Comparison with prior evidence*” compares the finding with previous literature, demonstrating how the narrative review integrates fragmented prior evidence, emphasizes cross-cutting themes, and identifies translational insights. It discusses how AI-enabled apps advance beyond traditional mHealth tools, highlighting gaps in prior secondary studies.

*Section 4.3* “*Exploring clinical and policy implications*” examines the real-world impact of AI-powered mobile health applications on clinical practice and healthcare policy. It highlights how these tools support continuous patient monitoring, early detection, and personalized interventions across chronic diseases, mental health, and preventive care. The discussion emphasizes the importance of integrating apps into clinical workflows, ensuring usability, and promoting patient engagement. Policy considerations focus on data privacy, equitable access, and addressing algorithmic bias. Overall, the subsection underscores that while AI-mHealth applications hold transformative potential, their benefits depend on careful implementation, governance, and alignment with healthcare priorities.

*Section 4.4* “*Exploring the regulatory landscape surrounding AI-powered mobile health apps*” explores the evolving regulatory environment governing AI-powered mobile health applications, highlighting various thematics in the found studies. This section provides an overview of relevant medical device regulations, including classification, approval processes, and post-market surveillance requirements. Additionally, it examines emerging AI-specific regulatory frameworks and guidelines that address algorithmic transparency, bias mitigation, and ethical considerations. By discussing the convergence and gaps between traditional medical device regulation and AI governance, this subsection highlights the regulatory challenges and opportunities critical for ensuring patient safety and fostering innovation.

*Section 4.5* “*Quality Aspects in AI & Apps*” focuses on quality aspects of AI-powered health apps. It synthesizes evidence on usability, clinical validity, reliability, and user experience, emphasizing how structured evaluation frameworks and evidence-based design principles contribute to app effectiveness and adoption. Key considerations include interoperability with clinical workflows, robustness of AI algorithms, adherence to clinical guidelines, and patient-centered design, all of which influence both user trust and regulatory acceptance.

*Section 4.6* “*Technical Standards and Norms for AI-Powered Health Apps*” addresses technical standards and norms applicable to AI-powered health apps. Compliance with ISO, IEC, and other relevant frameworks ensures that AI applications are safe, reliable, interoperable, and privacy-conscious. This subsection elaborates on standards covering software lifecycle management, risk mitigation, usability, data protection, AI governance, and post-market surveillance. By following these norms, developers and healthcare organizations can enhance algorithm transparency, mitigate bias, ensure continuous monitoring, and foster trust among clinicians, patients, and payers.

*Section 4.7* “*The Regulatory Gray Zone and AI-Specific Advantages in mHealth*” highlights the regulatory “gray zone” in mobile health, where many applications perform medical-like functions without formal classification or oversight, creating gaps in patient safety, liability, and governance. Examples include fitness or wellness apps versus regulated cardiac or mental health screening tools. At the same time, AI-specific advantages are discussed, such as real-time pattern recognition, personalized interventions, predictive modeling, and natural language processing for symptom assessment. The subsection underscores the need to balance innovation with safety, addressing both the regulatory ambiguities and the unique benefits AI brings to mHealth.

*Section 4.8* “*Limitations*” outline the study’s limitations. It addresses methodological constraints inherent in narrative reviews, such as potential selection bias and the lack of quantitative synthesis. The subsection also considers limitations related to the rapidly evolving nature of AI and mHealth technologies, which may impact the generalizability and timeliness of the findings.

*Section 4.9* “*Recommendations for Future Research*” addresses emerging recommendations and future research priorities for AI-powered mHealth applications. It focuses on rigorous evaluation of long-term clinical effectiveness, algorithm transparency and explainability, bias mitigation, adaptation of regulations for apps in the “gray zone,” integration with clinical workflows, healthcare system interoperability, and ethical considerations such as privacy, informed consent, and user engagement. The aim is to guide the safe, equitable, and sustainable development of AI in mobile health.

### 4.1. Summary of Key Findings

The overview of secondary studies reveals three major insights regarding the integration of AI in mHealth applications, encompassing growing contributions, emerging categorizations, and the balance of opportunities and challenges, thereby offering a comprehensive understanding of AI’s role in transforming healthcare delivery.

*First*, the integration of AI in mHealth apps has proven transformative across multiple healthcare domains, particularly in disease prevention, diagnosis, and management. AI technologies, including predictive algorithms for mental health management and machine learning models for chronic disease care, enhance healthcare quality by enabling real-time monitoring and tailored interventions. For instance, predictive AI tools in mental health care can anticipate symptom onset and provide early interventions, potentially preventing crises and improving long-term outcomes, especially among youth [21]. Similarly, AI applications in ophthalmology, such as the diagnosis of retinal diseases and diabetic retinopathy, demonstrate the potential for early detection and precise diagnostics, which are critical in preventive healthcare [30,43].

*Second*, several thematic patterns and emerging categorizations are evident across the literature. mHealth apps can be classified by functionality, including disease monitoring, diagnostic support, and behavior modification. AI’s impact is increasingly visible in personalized healthcare, with apps tailored to meet the specific needs of individuals with chronic conditions such as diabetes, hypertension, and cardiovascular diseases [24,35]. Preventive health remains a significant trend, with AI supporting early detection and risk assessment for conditions like cancer, cardiovascular disease, and sleep disorders. Tools for early disease detection, including oral cancer [23] and sleep apnea [47], illustrate the clinical relevance of AI in enabling timely interventions. Additionally, AI-driven behavioral health interventions, such as chatbots for smoking cessation and mental health support, showcase AI’s potential in addressing lifestyle-related health issues [28].

*Finally*, while AI integration offers substantial opportunities, it also presents notable challenges. AI-powered mHealth apps can improve healthcare accessibility, particularly for underserved or remote populations, and facilitate remote monitoring and diagnosis, reducing the need for in-person visits—a role that proved crucial during the COVID-19 pandemic [53,56]. Personalized interventions further enable tailored management of chronic conditions, potentially reshaping patient care [29,40]. However, challenges persist, including data privacy and security, technological accessibility in low-resource settings, regulatory complexities, and the risk of algorithmic bias that could produce inequitable outcomes [33].

Overall, the growing presence of AI in mHealth is reshaping healthcare by improving diagnostic accuracy, enabling personalized care, and supporting disease management. While opportunities are vast, addressing challenges such as data security, accessibility, and regulation is essential for equitable and effective integration. Continued efforts to overcome these barriers will determine whether AI can fully realize its potential to make healthcare more accessible, efficient, and personalized across diverse populations.

### 4.2. Comparison with Prior Evidence

This narrative review complements and extends prior work in mHealth research by focusing on emerging themes and clinical implications of AI integration, rather than following the strict inclusion criteria of secondary studies.

Earlier studies, such as Fiordelli et al. (2013) [6], mapped a decade of mHealth evolution, providing a broad overview of mobile health research but offering limited insight into the integration of AI in clinical practice. Hobson et al. (2019) [7] examined mHealth for First Nations populations, emphasizing accessibility and engagement, yet without detailing AI functionalities or clinical impact.

During the COVID-19 pandemic, the overviewed studies addressed mobile apps for pandemic management. Holl et al. (2024) [8] reviewed COVID-19 apps, focusing mainly on adoption and usage patterns. Mansouri & Darvishpour (2023) [9] provided a scoping review of reviews, again emphasizing usage and deployment rather than clinical outcomes or translational relevance.

Other studies specifically considered AI integration. Maccioni & Giansanti (2021) [10] discussed the “gray zone” of medical apps and the need for cybersecurity expansion. Dabla et al. (2021) [11] highlighted the emerging role of AI in mobile health and digital laboratory medicine. Alkasassbeh et al. (2023) [12] explored COVID-19 detection apps, emphasizing diagnostic potential. Shahroz et al. (2021) [13] examined digital contact tracing applications. Huang et al. (2022) [14] discussed telemedicine and AI to support self-isolation. Solimini et al. (2021) [15] analyzed ethical and legal challenges of telemedicine during COVID-19. Kritikos (2022) [16] addressed ethical governance of AI applications in pandemics. Lastrucci et al. (2024) [17] proposed algoretics frameworks for balancing innovation and integrity in healthcare AI. Singareddy et al. (2023) [18] reviewed AI for chronic condition management. Zhang et al. (2024) [19] offered a global perspective on AI in telemedicine.

While these works provide valuable insights, many remain fragmented, emphasizing either technical aspects, pandemic-specific applications, or ethical and regulatory considerations in isolation. They often do not systematically connect technological innovations to clinical relevance and cross-cutting themes in mHealth.

In contrast, this narrative review synthesizes a wide spectrum of evidence to identify recurring themes and emerging trends across healthcare domains. AI-driven mHealth tools are increasingly applied to personalized care, chronic disease management, mental health interventions, and preventive diagnostics [21,24,28,30,35,43,47]. This approach highlights patterns that transcend individual studies or populations, capturing innovation trends that systematic reviews with narrower scopes might overlook.

Moreover, this review incorporates insights on regulatory, ethical, and implementation aspects, including medical device classification, data standardization, privacy, and cybersecurity [26,31,33,39,57,64,69,70,71,72,73,74,75,76]. By maintaining a narrative perspective, the review captures the breadth of the field, linking technological advances to patient care and highlighting opportunities and challenges for both research and policy.

In summary, while systematic reviews provide rigorous but narrowly defined evidence, this narrative synthesis identifies cross-cutting themes, translational insights, and areas of emerging innovation, reflecting the dynamic and evolving landscape of AI-powered mHealth.

### 4.3. Exploring Clinical and Policy Implications

The integration of Artificial Intelligence (AI) into mHealth apps carries profound implications for both clinical practice and healthcare policy. Successful implementation requires careful consideration of several interconnected domains to ensure that AI technologies are safe, effective, and equitable.

*Medical Device Classification and Integration*

AI-driven mHealth apps frequently perform functions akin to medical devices, including diagnostic support for chronic diseases such as diabetes and hypertension, or conditions like diabetic retinopathy [30,35]. To ensure safe clinical use, these tools must comply with stringent medical device regulations, including validation through clinical trials that assess accuracy, safety, and efficacy. Establishing reliability through evidence-based validation helps address concerns regarding AI performance [26,32]. Recent studies emphasize the urgency for adaptive regulatory frameworks that keep pace with rapidly evolving AI technologies [57,63].

*Standardization of Data and Tools*

AI implementation depends heavily on data quality and interoperability. Variability in data collection and platform standards can compromise the performance of AI algorithms, especially in multi-center or multi-region settings. Standardized data protocols, unified AI models, and platform interoperability are critical for consistent, reliable, and scalable deployment. Several studies highlight that achieving standardization is essential for effective chronic disease management, diagnostics, and other AI applications [24,28,64,69].

*Ethical Considerations*

The ethical dimension of AI in healthcare encompasses transparency, explainability, bias mitigation, and patient autonomy [31,33]. Patients must be informed about AI-driven interventions and consent to their use, particularly in mental health or chronic disease management, where algorithmic bias could exacerbate inequities. Developers must prioritize fairness and patient-centered design to prevent disparities and promote trust in AI technologies. Emerging evidence underscores the importance of robust ethical frameworks to ensure equitable AI deployment [70,71,72,73].

*Cybersecurity and Data Privacy*

Protecting sensitive health data is essential when deploying AI-powered mHealth apps [21,39]. Robust cybersecurity measures—including encryption, secure storage, and regular security audits—are necessary to safeguard patient information. Privacy-preserving approaches, such as federated learning, allow AI models to be trained without centralized data sharing. Compliance with privacy regulations, including GDPR and HIPAA, is critical to maintain patient trust and avoid breaches [74,75,76].

*Regulation and Approval Processes*

Regulatory oversight ensures that AI tools are safe, effective, and clinically appropriate. Current approval pathways vary across regions, creating challenges for widespread adoption [57,61,68]. Streamlined processes that retain rigorous evaluation standards are needed to accelerate clinical integration [59,65]. Thorough validation through clinical trials and quality assessment is essential before AI-powered tools can be widely implemented [26,50,72].

In summary, these domains—device classification, data standardization, ethics, cybersecurity, and regulatory processes—are deeply interconnected [63,67,74]. Holistic attention to all areas is crucial to ensure that AI in mHealth applications is safe, equitable, and effective. Robust cybersecurity protects patient data, regulatory oversight ensures safety and fairness, ethical frameworks guide transparency and consent, and standardized data protocols enhance reliability and interoperability [58,60,62,64,66,69,70,71,73,75,76]. Addressing these interconnected challenges lays the foundation for AI-driven transformations in patient care, clinical workflows, and healthcare system efficiency. As AI technologies mature, the demand for comprehensive and adaptive regulatory frameworks becomes increasingly urgent.

### 4.4. Exploring the Regulatory Landscape Surrounding AI-Powered Mobile Health Apps

#### 4.4.1. Characteristics of the AI-Based App Concerning Medical Device Regulation

Mobile health apps marketed as medical devices fall under the definition of a software medical device (SaMD), as first introduced by the International Medical Device Regulators Forum in 2013 [77]. Currently, there are several standards for managing SaMDs [78,79,80,81,82], but there are still no fully harmonized, AI-specific regulations exclusively dedicated to AI-based SaMDs. However, guidelines can be found on the FDA site [83], and some standards are under development [84]. SaMDs including AI (Machine Learning and/or Deep Learning) offer huge potential, but they also come with significant risks that regulators, developers, and healthcare providers must manage carefully. The greatest risks relate to

- Transparency of algorithms (known as ‘black boxes’), which do not allow clinicians to understand or verify the decisions made by the algorithm;
- Effectiveness, since unlike traditional software, artificial intelligence systems can change their behaviour over time, which can lead to unnecessary treatments or missed diagnoses;
- Depending on the input data, there is a risk of bias if the training data has not been diversified;
- Data security and privacy, as there is a risk of accidental data loss and cyberattacks;
- Regulatory and legal uncertainty, as the regulatory frameworks of various countries are still evolving.

We searched, with the string in Table S3, *position 1*, for artificial intelligence-powered mobile health apps marked as a medical device (e.g., CE-marked (Conformitè Europeenne) or FDA-approved). Studies that referred to the keywords were included and analysed: 30 studies were found from this search, but only 3 studies presented certified medical devices, and all three devices found were CE-marked. The regulation for CE-marked medical devices is the Regulation (EU) 2017/745 (MDR) [85]. Medical devices are classified based on the risk they pose to patients and users. Different countries and regulatory authorities use these classification systems to determine the level of control necessary to ensure safety and effectiveness. Risk classes are denoted by increasing numbers that correspond to the level of risk. Low risk implies less regulatory control, while high risk implies more rigorous oversight. In the European Union (CE mark) medical devices are classified into four main classes: Class I (low risk; non-invasive products self-certified by manufacturers); Class IIa (low to medium risk; requires notified body involvement); Class IIb (medium to high risk; more scrutiny by notified bodies); Class III (high risk; requires comprehensive review and clinical evaluation).

To analyze some characteristics of the devices reported in the three selected studies [86,87,88], we searched for them in the European Database on Medical Devices, named Eudamed (https://ec.europa.eu/tools/eudamed/#/screen/home, accessed on 28 July 2025) and in the Italian Database of medical devices (https://www.salute.gov.it/interrogazioneDispositivi/RicercaDispositiviServlet?action=ACTION_MASCHERA, accessed on 28 July 2025). Registration in the European database is currently not compulsory, whereas it is compulsory for devices marketed in Italy. For the European market, manufacturers register medical devices in the EUDAMED database with a code according to the European Nomenclature of Medical Devices. This nomenclature derived from the Italian National Classification of Medical Devices (Classificazione Nazionale dei Dispositivi Medici-CND: https://www.salute.gov.it/new/it/tema/dispositivi-medici/la-classificazione-nazionale-dei-dispositivi-medici-cnd/, accessed on 28 July 2025), which groups medical devices into homogeneous categories of products intended to perform a similar diagnostic and/or therapeutic intervention. Table 6 below summarizes the characteristics of the studies [86,87,88] and medical devices presented.

#### 4.4.2. Characteristics of the AI-Based App Concerning AI Regulation

Many countries do not yet have comprehensive AI regulations, but there are numerous regulatory frameworks and guidelines that define basic principles for developing AI solutions. The AI regulatory frameworks in the Western world (including most of Europe, UK, US and Canada, and Australia and New Zealand) [89,90,91,92,93] are based on basic principles derived from the OECD AI principles (OECD: Organisation for Economic Co-operation and Development), designed to help ensure that AI is trustworthy and ethically sound (https://www.oecd.org/en/topics/ai-principles.html, accessed on 28 July 2025). These principles can be summarized as follows:

1. Robustness and safety: AI systems must operate reliably and safely.
2. Privacy and security: AI systems must respect privacy (mandatory information on how data are collected, used, and stored.) and must be secure (system access and data encryption to ensure data quality and integrity).
3. Transparency and explainability: AI operations must be understandable to users and stakeholders, from training on the data and algorithms to generating the final model.
4. Fairness and inclusiveness: AI outcomes must avoid bias and discrimination toward or against certain groups of people.
5. Accountability: There must be clear responsibilities for the results of the AI system, such as to the developers who design and deploy the AI system.

We searched for studies on AI-powered mobile health apps that also dealt with regulatory issues regarding artificial intelligence (see the string in the Table S3, position 2). We found 52 studies including app studies and review studies. Nine studies [94,95,96,97,98,99,100,101,102] were relevant to our research and are summarized in Table 5.

The AI regulatory issues discussed in the analyzed studies are shown in the middle column of Table 7, “Regulatory Issue”. According to the schematization of the basic principles of AI described above, these issues were considered to belong to one or more of the basic principles.

Most of the comments on the use of AI concerned the need for regulation in the areas of reliability (of clinical decisions), accountability of the clinical act (especially in the case of incorrect decisions), quality assurance and data security.

Although there is still no comprehensive and harmonized regulatory framework specifically tailored to AI-based medical devices, and only a few guidelines exist [83,84], many devices have already been authorized between 2015 and 2023, as reported in a recent review [103]. The review provides data on AI-based software as a medical device (also defined as AI-SaMD) approved by the FDA [104]. It should be noted that in Europe, a recent FAQ document [105] links the regulation of medical devices with the European CE mark to the AI Act. This document applies to SaMD with a CE mark, such as those identified in the studies reviewed here. This guidance has been endorsed by the Artificial Intelligence Board (Article 65 of the AI Act Regulation (EU) 2024/1689 [91]), which is a coordinating and advisory body responsible for the implementation of AI, and the Medical Device Coordination Group (MDCG; Article 103 of Regulation (EU) 2017/745 [85]), which is an expert committee assisting the European Commission and the Member States in ensuring harmonised implementation of medical device regulations. This guidance uses the acronym MDSW to define medical device software and proposes a new definition for AI systems intended for medical use: “Medical Device Artificial Intelligence” (MDAI). Although it is not binding, the guidance can serve as a reference for medical device software manufacturers, consultants, and notified bodies, helping them to bring safe, effective and robust AI-based medical device software to market.

### 4.5. Quality Aspects in AI & Apps

Ensuring high-quality AI-powered mHealth applications requires a multidimensional and integrative approach, encompassing policy alignment, clinical efficacy, economic impact, usability, and structured assessment frameworks. Quality is not a single attribute but a composite of safety, effectiveness, reliability, data integrity, privacy, transparency, and user trust, which together determine the value, acceptability, and adoption of digital health technologies.

High-quality apps are expected to support clinical decision-making, enhance patient self-management, facilitate care coordination, and provide actionable insights while minimizing risks such as misdiagnosis, data breaches, or low adherence. Achieving these objectives requires a holistic view, integrating technological robustness with human factors, regulatory compliance, and alignment with healthcare system priorities.

#### 4.5.1. Policy and Global Strategies

Global strategies provide the foundation for the development, deployment, and evaluation of AI-driven health apps. The World Health Organization’s Global Strategy on Digital Health 2020–2025 emphasizes evidence-based innovation, equitable access, interoperability, and alignment with health system priorities [106]. These principles aim to guide policymakers, developers, and healthcare organizations toward technologies that enhance health outcomes, reduce disparities, and foster sustainable digital ecosystems. Importantly, global strategies highlight the need to monitor real-world impact, ensuring that interventions translate into measurable health improvements.

Similarly, Butcher and Hussain [107] emphasize the transformative potential of digital health solutions to redefine care delivery, with AI-enabled apps supporting earlier diagnosis, remote patient monitoring, predictive risk stratification, and preventive interventions. Deloitte’s 2019 report [108] and IQVIA’s 2021 trends report [109] further contextualize these developments, identifying emerging technologies, regulatory considerations, and adoption drivers that shape the global digital health landscape. Collectively, these frameworks provide guidance on ethical development, alignment with clinical pathways, and prioritization of user-centered design, helping to ensure that AI solutions are not only innovative but socially and clinically responsible.

#### 4.5.2. Clinical Evidence and Effectiveness

Clinical effectiveness is central to app quality. AI-powered apps can offer personalized interventions, continuous monitoring, and predictive analytics, potentially transforming both preventive and chronic care. Evidence from randomized controlled trials (RCTs) and secondary studies supports measurable benefits across multiple domains:

- Mental health interventions: Apps delivering mindfulness, cognitive behavioral therapy (CBT), or stress reduction programs improve well-being, reduce anxiety and depressive symptoms, and enhance coping skills [110,111]. Integration of AI-driven personalization allows dynamic adaptation of content, reminders, and exercises based on individual user progress, engagement patterns, and risk profiles.
- Chronic disease management: Diabetes, hypertension, and cardiovascular apps facilitate lifestyle modification, medication adherence, and biometric monitoring, demonstrating improvements in glycemic control, blood pressure, and symptom management [112,113,114]. AI models can identify early deviations from expected health parameters, alert patients and clinicians, and recommend timely interventions.
- Oncology care: Mobile apps support cancer survivors in self-management, symptom tracking, and psychological support, improving quality of life and reducing stress [115,116]. Predictive analytics enable anticipation of side effects or disease progression, allowing personalized follow-up schedules and care adjustments.
- Respiratory disease support: Asthma and COPD apps enhance adherence, track inhaler usage, and provide environmental alerts [117]. AI personalization can optimize medication timing, offer actionable recommendations, and support behavior change interventions to reduce exacerbations.

Effectiveness depends not only on algorithmic accuracy but also on user engagement, clinical integration, and contextual appropriateness, highlighting the need for iterative testing and real-world validation.

#### 4.5.3. Economic Evidence and System-Level Impact

The quality of AI-powered apps is also assessed in economic terms, including cost-effectiveness, system efficiency, and scalability. Digital health interventions can reduce hospital admissions, optimize resource allocation, and improve adherence, particularly in chronic care pathways [118,119].

Socio-economic analyses demonstrate that mHealth solutions can reduce healthcare inequalities by increasing access to underserved populations, improving self-management capabilities, and mitigating geographic or socioeconomic barriers to care [120]. Economic evaluation supports informed reimbursement and funding decisions, ensuring that high-quality apps are both clinically beneficial and financially sustainable.

Integration of economic evidence into quality frameworks allows policymakers and healthcare organizations to prioritize interventions with high health impact per cost, aligning innovation with system-level goals and population health priorities.

#### 4.5.4. Adoption and Usability

Even clinically effective and cost-efficient apps fail if they are not adopted by patients and healthcare professionals. Adoption depends on a complex interplay of technical usability, trust, workflow integration, and perceived value:

- Clinician adoption: Healthcare providers consider usability, evidence of clinical effectiveness, integration with electronic health records, and compatibility with existing workflows [121]. AI recommendations must be transparent, interpretable, and explainable to gain trust and support clinical decision-making.
- Patient uptake: Sociotechnical factors—such as digital literacy, engagement strategies, trust in data handling, and behavioral design—affect sustained use [122]. Personalized notifications, gamification, and interactive feedback can improve adherence.
- Quality perception vs. popularity: App store ratings, downloads, or user reviews often do not correlate with clinical quality or safety, underscoring the need for structured assessment [123,124].
- Quality labeling: Certification schemes and evidence-based quality labels can enhance user trust, professional recommendation, and uptake, providing an objective signal of safety, effectiveness, and reliability [125,126].

Fostering adoption requires a user-centered design approach, continuous feedback loops, and ongoing support for both clinicians and patients.

#### 4.5.5. App Assessment Frameworks and Standardization

Standardized assessment frameworks provide objective criteria for evaluating app quality, including functionality, reliability, clinical evidence, data privacy, interoperability, and user experience.

- European mHealth Hub: Offers comprehensive guidelines to assess app features, clinical validation, usability, and cybersecurity [127].
- CEN ISO/TS 82304-2: Internationally recognized criteria cover safety, effectiveness, interoperability, engagement, and transparency, supporting consistent and reproducible evaluation [128].
- Iterative testing and reliability: Pilot studies validate frameworks, ensuring robustness and applicability across diverse healthcare contexts [129,130].

Standardization enables comparisons across apps, informs regulatory and reimbursement decisions, guides continuous improvement, and ultimately strengthens user confidence in digital health solutions. The integration of policy, clinical evidence, economic assessment, and usability creates a holistic framework for defining and ensuring quality in AI-powered mHealth applications.

### 4.6. Technical Standards and Norms for AI-Powered Health Apps

AI-powered health applications operate at the intersection of digital medicine, software engineering, and clinical care. Ensuring their safety, effectiveness, interoperability, and data integrity requires adherence to technical standards and norms. Given the vast number of ISO, IEC, and other guidelines, this section focuses on a selection of standards considered most relevant for AI-powered health apps. Readers are referred to specialized texts for a more comprehensive overview.

#### 4.6.1. ISO and IEC Standards

ISO and IEC standards provide internationally recognized guidance for software quality, safety, and risk management, with specific relevance to health applications. Compliance with these standards helps developers ensure that apps are robust, safe for patients, and aligned with regulatory expectations. For AI-powered health apps, they also support governance of algorithmic decision-making and lifecycle management. The following Table 8 summarizes the selected ISO and IEC standards applicable in this context.

#### 4.6.2. Health IT and Interoperability Standards

Interoperability and information security are critical when AI-powered apps exchange data with healthcare IT systems or medical devices. Standards in this area define technical and organizational requirements for secure communication, device integration, and data protection, which are essential for ensuring safe clinical workflows and patient privacy. Table 9 below lists the most relevant interoperability and IT standards.

#### 4.6.3. AI-Specific Guidelines and Regulatory Alignment

AI introduces unique challenges in healthcare, including algorithm transparency, bias, model validation, and adaptive learning. Dedicated AI guidelines provide frameworks for responsible design, clinical validation, and ethical deployment, complementing general software and medical device standards. Table 10 below presents the most relevant AI-specific guidance for health apps.

#### 4.6.4. Quality Assessment, Certification, and Continuous Monitoring

Continuous monitoring and quality assessment are essential to maintain clinical reliability, regulatory compliance, and user trust over time. Standards in this domain define evaluation frameworks, certification criteria, and mechanisms for iterative improvement of apps and AI models. The following Table 11 summarizes the key standards supporting quality assessment and continuous monitoring.

### 4.7. The Regulatory Gray Zone and AI-Specific Advantages in mHealth

#### 4.7.1. The Regulatory Gray Zone in mHealth Applications

A recurring challenge in the digital health landscape is the regulatory gray zone, referring to applications that perform functions resembling medical devices but lack formal classification or regulatory oversight [10,77,78]. These are apps that, while capable of influencing health outcomes, are not clearly regulated under existing frameworks such as the FDA guidance on Software as a Medical Device (SaMD) [78] or the European Union Medical Device Regulation (MDR 2017/745) [82,85].

This gray zone can be illustrated with several examples. On one end of the spectrum, fitness apps track steps, heart rate during exercise, or general activity levels. These tools are typically unregulated, as they provide general wellness information and do not claim to diagnose or treat diseases. Similarly, wellness or mindfulness apps offer guided meditation, stress tracking, or breathing exercises without formal clinical validation [110,111].

In contrast, cardiac monitoring apps that analyze electrocardiogram (ECG) data to detect arrhythmias are often regulated as Class II medical devices in the United States, requiring clinical validation and regulatory clearance [77,79]. Likewise, apps designed for clinical depression screening or mental health diagnostics can fall under SaMD regulations, depending on the claims made about diagnosis or treatment recommendations [98,102].

The implications of this gray zone are significant. First, patient safety risks may arise when apps provide diagnostic insights without validated accuracy, potentially delaying proper care or causing mismanagement of conditions. Second, liability gaps exist because the legal responsibility for unregulated app outputs is often unclear—neither developers nor healthcare providers may be fully accountable. Third, this situation emphasizes the urgent need for governance and guidance frameworks that clarify classification criteria, encourage voluntary certification, and support safe integration of AI-based mHealth tools into clinical practice [82,91,105].

Efforts to address the gray zone are underway. The FDA has issued guidance on SaMD, clarifying when software requires regulatory oversight [78,79], and the MDCG guidance in the EU provides frameworks for qualifying software under MDR/IVDR [82,105]. Additionally, the EU Artificial Intelligence Act (2024/1689) introduces risk-based categorization for AI-driven health applications, further shaping the regulatory landscape [91]. Despite these initiatives, many consumer-facing apps remain in the gray zone, highlighting a critical need for continuous monitoring, standardized quality labeling, and public awareness.

#### 4.7.2. AI-Specific Advantages vs. Traditional mHealth Tools

While traditional mHealth applications provide tracking, reminders, and basic educational content, AI integration introduces distinct advantages that can substantially enhance healthcare delivery. These AI-specific capabilities extend beyond simple monitoring and enable personalized, predictive, and adaptive interventions.

1. Real-time Pattern Recognition and Anomaly Detection
  AI algorithms can analyze continuous streams of patient data to detect subtle anomalies that human observers or traditional apps may miss. For example, in diabetes management, AI-enabled apps can monitor glucose trends and detect patterns predicting hyperglycemia or hypoglycemia, allowing for early interventions [112,114]. Similarly, AI can identify early signs of cardiac arrhythmias from wearable ECG data, outperforming rule-based alerts provided by standard apps.
2. Personalization Based on Individual Data
  Unlike conventional mHealth tools, AI can tailor interventions to each user’s unique physiology, behavior, and health history. In oncology, AI-driven apps can provide individualized medication reminders and lifestyle recommendations for breast cancer survivors [115,116]. In mental health, AI chatbots can adapt counseling content based on real-time mood assessments, optimizing engagement and efficacy [96,98].
3. Predictive Modeling and Risk Stratification
  AI supports predictive analytics, allowing clinicians and patients to anticipate future health events. For example, AI tools can stratify cardiovascular risk by integrating patient demographics, lab results, and lifestyle data, guiding preventive interventions [115,117]. Predictive modeling can also prioritize high-risk patients for early monitoring, reducing hospitalizations and healthcare costs.
4. Natural Language Processing for Symptom Assessment
  AI-powered natural language processing (NLP) enables apps to interpret unstructured patient-reported information. Chatbots and digital symptom checkers can understand patient inputs in free text, detect critical symptoms, and triage patients appropriately [96,98]. This functionality is particularly valuable in mental health, where subjective symptom reporting is crucial.

However, AI is not universally superior. Traditional mHealth approaches remain advantageous in scenarios where simplicity, interpretability, and cost-effectiveness are critical. For instance, basic reminder apps, lifestyle trackers, or educational platforms are easier to implement, require minimal technical infrastructure, and are more transparent to users. Therefore, the integration of AI should be strategic, complementing rather than replacing conventional tools, particularly in resource-limited settings.

#### 4.7.3. Implications for Practice and Policy

Understanding both the regulatory gray zone and AI-specific advantages is essential for clinicians, developers, and policymakers. Clinicians must critically evaluate app claims, considering whether AI features are evidence-based and whether the app is regulated. Developers should aim for transparency, validation, and alignment with regulatory standards. Policymakers should clarify classification criteria, incentivize certification, and support frameworks that enable safe and equitable access to AI-driven mHealth tools [10,77,83,91,105].

Taken together, addressing the gray zone while leveraging AI-specific advantages can ensure that mHealth innovations are safe, effective, and impactful, transforming patient care while minimizing risks. This approach highlights the need for integrated regulatory, technical, and ethical strategies as AI continues to expand its role in digital health.

### 4.8. Limitations

This narrative review was chosen to provide a broad and flexible synthesis of a fast-evolving field, where rigid systematic reviews may inadvertently exclude emerging insights due to strict inclusion criteria. While systematic reviews offer rigorous evidence synthesis, they often impose tight constraints that can limit the scope of rapidly developing research areas like AI in mobile health.

Focusing on secondary studies allowed us to capture the latest developments and critical reflections shaped by recent technological and clinical advances. Importantly, these recent secondary studies generally build upon, update, and integrate findings from earlier studies, ensuring that no significant foundational evidence is overlooked. In this way, the narrative review benefits from a comprehensive evidence base while emphasizing current trends and practical applications.

We acknowledge that by restricting the analysis to peer-reviewed English-language literature and higher-level secondary studies, some evidence from non-English-language sources and emerging primary studies may have been excluded, introducing potential language and structural bias. However, we have corroborated and contextualized these findings in a differential and complementative manner in the discussion, incorporating additional scientific products, articles, and documents.

Furthermore, the fast-paced evolution of AI and mobile health technologies poses inherent challenges to the generalizability and long-term applicability of our findings, as new innovations may quickly outdate current evidence.

Despite these constraints, this narrative review offers a valuable, timely overview that balances methodological rigor and interpretative flexibility, making it particularly suitable for monitoring innovation and guiding clinical practice in a swiftly changing domain.

### 4.9. Recommendations for Future Research

The landscape of AI-driven mHealth applications is rapidly evolving, yet our review underscores persistent gaps in evidence, regulatory clarity, and practical implementation that must guide future research. A particularly pressing issue is the regulatory “gray zone,” where many AI-enabled apps perform functions closely resembling medical devices but lack formal classification or oversight [10,77,83,91,105]. For instance, fitness apps that track activity levels or wellness apps offering mindfulness exercises are often unregulated, whereas cardiac monitoring or clinical depression screening applications are subject to rigorous classification as Class II or higher devices under FDA or European MDR frameworks [77,78,82,83]. This regulatory ambiguity creates risks for patient safety, clinician liability, and public trust, and may slow the adoption of potentially beneficial technologies. Comparative research across jurisdictions and regulatory frameworks could clarify best practices and inform guidelines for safe, responsible, and equitable deployment.

In addition, as shown in Figure 8, the 56 selected reviews reveal a strong concentration of research in China, the United States, and Canada, with limited contributions from other regions. This geographical skew highlights a critical need to expand the scope of studies to include low- and middle-income countries, under-resourced settings, and areas with diverse healthcare infrastructures and regulatory environments. Incorporating broader geographical representation will improve the generalizability of findings, enable context-sensitive implementation strategies, and ensure equitable access to AI-driven mHealth solutions globally. International collaboration, capacity-building, and knowledge-sharing initiatives should be prioritized to support responsible development and deployment in diverse contexts.

Another priority is empirically evaluating the unique advantages of AI compared to traditional mHealth tools. While AI can offer real-time pattern recognition, predictive modeling, personalized recommendations, and natural language processing for symptom assessment [21,24,28,30,35,43,47], the evidence for tangible improvements in patient outcomes or healthcare efficiency is still limited. Future studies should implement comparative designs, directly contrasting AI-driven interventions with standard apps. For example, predictive AI tools in mental health management, such as mood forecasting apps, could be systematically compared to conventional symptom trackers or self-report diaries to assess the added value in early intervention and crisis prevention [21,28,110,111]. Similarly, in chronic disease management, AI-based diabetes or cardiovascular monitoring platforms should be evaluated against traditional self-management apps to quantify improvements in adherence, clinical outcomes, and patient engagement [24,35,112,114,119].

Data quality and standardization emerge as central determinants of AI performance. Variability in data sources, measurement methods, and interoperability across platforms can undermine the accuracy and generalizability of AI models [83,105]. Future research should explore strategies for harmonizing datasets, improving representativeness, and enabling cross-platform integration, particularly in multicenter or multinational studies. For instance, studies on AI-assisted pulmonary rehabilitation in COPD or mobile self-management interventions for cancer survivors demonstrate that data heterogeneity can limit the transferability of predictive models and affect the reliability of clinical decision support [115,116,117].

Ethical considerations and equity issues also demand systematic investigation. Algorithmic bias arising from unrepresentative datasets, design flaws, or unintended demographic skew can lead to inequitable outcomes, particularly for vulnerable populations [31,33,83,94,95,96,98]. Future research should rigorously evaluate bias mitigation strategies, transparency mechanisms, and patient-centered consent processes. Integrating privacy-preserving technologies, such as federated learning, could enable large-scale AI training without compromising sensitive health information, a concern highlighted in recent studies on dermatology and mental health apps [95,97,98]. Ensuring that AI tools are inclusive, transparent, and accountable will be essential for ethical deployment at scale.

The implementation and integration of AI-mHealth apps in real-world clinical workflows remain underexplored. Evidence from controlled trials often fails to capture barriers such as usability, adherence, workflow disruption, and long-term patient engagement [114,117,118,119]. Implementation science frameworks should guide future studies to identify factors that promote sustainable adoption, evaluate cost-effectiveness, and assess health system impacts. Multidisciplinary collaboration involving clinicians, data scientists, ethicists, and regulatory bodies will be crucial to co-design interventions that are both effective and contextually appropriate [10,91].

An additional research recommendation concerns the development of explainable AI within web-based and app-delivered mHealth environments. Recent studies have shown that AI systems deployed through user-facing software platforms can combine high diagnostic or predictive performance with interpretable outputs by integrating explainable machine learning techniques [141,142]. Although these approaches are often grounded in image- or sensor-based data streams, their web-based implementation highlights a transferable model for mHealth applications approaching diagnostic or decision-support functions. Future research should therefore explore how explainability, transparency, and clinician-facing interpretability can be systematically embedded into AI-powered mHealth apps, particularly to support trust, regulatory alignment, and responsible adoption when clinical risk increases.

Finally, continuous monitoring and post-market evaluation are vital. AI algorithms and mobile platforms evolve quickly, and long-term evidence on safety, efficacy, and user outcomes is sparse [77,83,86,91,105]. Studies should track real-world performance, patient-reported outcomes, and potential adverse events to ensure ongoing reliability and effectiveness. This will not only strengthen the evidence base for AI-mHealth interventions but also inform iterative improvements, regulatory updates, and evidence-based policymaking.

Overall, future research should adopt a comprehensive, multi-level approach, encompassing regulatory clarification, comparative effectiveness studies, data standardization, ethical oversight, real-world implementation, and continuous monitoring. By addressing these interconnected priorities, researchers can ensure that AI-driven mHealth tools achieve their potential to improve healthcare delivery, patient outcomes, and health equity, while mitigating risks associated with unregulated innovation.

## 5. Conclusions

AI-powered mHealth technologies hold transformative promise for enhancing healthcare delivery through improved diagnostics, personalized interventions, and expanded access to care, especially in underserved and remote populations. These technologies enable real-time monitoring, early detection of disease exacerbations, and tailored treatment plans, which collectively improve patient outcomes and healthcare system efficiency. Moreover, by empowering patients with tools for self-management and continuous health tracking, AI-driven mHealth solutions foster greater patient engagement and autonomy.

However, harnessing this potential requires a concerted and multidisciplinary focus on overcoming key challenges related to data quality, ethical considerations, privacy protection, and regulatory oversight. Ensuring the accuracy and reliability of AI algorithms demands rigorous validation on diverse and representative datasets to prevent biases and inaccuracies that could compromise patient safety. Ethical transparency, explainability, and fairness must be embedded throughout the development and deployment of AI tools to maintain trust among patients and clinicians alike.

Privacy and cybersecurity are equally critical, given the sensitive nature of health data handled by these technologies. Robust measures including encryption, secure data storage, and adherence to data protection regulations such as GDPR and HIPAA are essential to safeguard patient information and maintain user confidence.

As AI solutions become increasingly integrated into clinical practice, the imperative for harmonized, stringent, yet flexible regulatory frameworks has never been greater. Current regulatory approaches must evolve to accommodate the adaptive and continuously learning nature of AI algorithms without compromising safety or efficacy. Global coordination among regulatory bodies is necessary to streamline approval processes, reduce fragmentation, and facilitate equitable access to these innovations worldwide.

Only through such coordinated efforts—encompassing technological innovation, rigorous validation, ethical governance, and regulatory harmonization—can we ensure that AI-powered mHealth technologies translate into safe, effective, and equitable healthcare for all. The future of healthcare depends on our collective ability to responsibly harness AI’s potential, balancing innovation with patient-centric values and societal needs.

## Figures and Tables

**Table 1:** Search terms and focus areas for AI in mHealth.

Focus Area	Mobile Health Terms	AI Terms	Notes
General AI in mHealth	“mobile health”, “mHealth”, “app *”, “smartphone”	“artificial intelligence”, “AI”	Broad capture of AI applications in mHealth
Machine Learning applications	“mobile health”, “mHealth”, “app *”, “smartphone”, “wearable devices”	“machine learning”, “ML”, “supervised learning”, “unsupervised learning”	Predictive analytics and automated decision-making
Deep Learning & Neural Networks	“mobile health”, “mHealth”, “app *”, “smartphone”	“deep learning”, “neural networks”, “CNN”, “RNN”	Advanced AI algorithms for clinical or monitoring purposes
Telemedicine & Remote Monitoring	“mobile health”, “mHealth”, “app *”, “smartphone”, “telemedicine”, “remote monitoring”	“AI”, “machine learning”, “deep learning”, “neural networks”, “CNN”, “RNN”, “predictive analytics”, “decision support”	Enhancing patient management and chronic disease monitoring
Specific Clinical Domains	“mobile health”, “mHealth”, “app *”, “smartphone”, “mobile apps”	“AI”, “machine learning”, “deep learning” + disease terms (e.g., “diabetes”, “cardiovascular disease”, “mental health”)	Targeted retrieval for specific disease areas

**Table 2:** Inclusion and Exclusion Criteria.

Criterion	Definition	Inclusion	Exclusion	Notes
Study Type	Type of publication	Reviews, with priority to Systematic reviews and Meta-analyses	Original research, Case reports, Technical notes	Ensures high level of evidence
Clinical/Bioengineering Relevance	Application in health or bioengineering	mHealth interventions with AI for chronic disease management, telemedicine, mental health, rehabilitation	Purely computational studies, algorithm benchmarking, data architecture without clinical/translational outcomes	Focus on real impact in clinical practice
Temporal Scope	Publication period	Studies up to 31 July 2025	Studies outside this period	Updated post-pandemic coverage
Language	Language	English	Non-English	International accessibility and reproducibility
Study Focus	Main content	AI applications with tangible clinical/translational value	Technical-only AI methods without healthcare relevance	Maintains focus on clinical narrative

**Table 3:** Sketch of the selected studies.

Ref.	Description	Focus	Contribution of mHealth and AI	Target Domain	Observed Patterns/Trends
[21] Patel J et al.	This review evaluates predictive artificial intelligence approaches used in mobile health platforms to forecast mental health symptoms among youth. It synthesizes how smartphone and wearable data, such as usage patterns, sleep, and physical activity, have been leveraged in predictive models to detect depression, anxiety, and stress. The study highlights both the promise of precision prediction and challenges including small sample sizes and privacy concerns that should be addressed in future work.	Predicting mental health symptoms (e.g., depression, anxiety, stress) using AI and mHealth tools.	Real-time monitoring of mental health; supports precision prevention strategies	Mental health (youth, 13–25)	Predictive AI models; mental health monitoring; youth-focused interventions
[22] Manyazewal T et al.	This study reviews innovative technologies, including AI and mobile health solutions, aimed at addressing neglected tropical diseases in African settings with ongoing sociopolitical instability. It discusses how these tools support disease surveillance, diagnosis, and intervention in contexts with limited healthcare infrastructure. The review emphasizes feasibility and adaptation of technological approaches in unstable and low-resource environments.	Use of AI, mobile apps, and other technologies for NTD prevention, diagnosis, and management amidst instability.	Supports NTD surveillance and treatment; AI aids in outbreak prediction and intervention planning	Neglected tropical diseases	AI for outbreak prediction; mHealth for disease management; low-resource and unstable regions
[23] Thakuria T et al.	This review examines the use of deep learning for early diagnosis of oral cancer via smartphone and DSLR image analysis. It highlights the role of convolutional neural networks in improving classification accuracy for early detection tasks. The study also discusses challenges such as data quality and limited annotated datasets, while noting the potential for accessible screening tools in low-resource settings.	Deep learning (CNN) applications for early oral cancer diagnosis using smartphone and DSLR imaging.	AI-based tools improve diagnostic accuracy; enhances accessibility of screening	Oral cancer diagnosis	Deep learning for image-based diagnosis; diagnostic accuracy improvements; potential for low-resource deployment
[24] Förstel M et al.	This review investigates features used in machine learning models to forecast patient arrival numbers in healthcare settings. It focuses on various predictor types, including non-temporal data such as weather and internet usage patterns, to enhance forecasting accuracy. The study highlights how these models can support operational planning, reducing prediction errors and improving resource allocation in clinical environments.	Machine learning feature identification for forecasting patient arrivals using non-temporal predictors.	ML forecasting aids operational planning; highlights value of diverse non-temporal features	Healthcare operations	Operational optimization via ML; non-clinical healthcare AI use; feature exploration beyond temporal data
[25] Chau RC et al.	This review discusses the potential of self-monitoring oral health using smartphone selfies powered by artificial intelligence for preventive dentistry. It evaluates how AI-enabled oral health tools can detect dental conditions and support early prevention by reducing plaque and gingival indices. The synthesis notes positive short-term outcomes and suggests areas requiring further research on long-term behavioral change and oral health awareness.	AI-enhanced self-monitoring of oral health via smartphone images and selfies.	Supports early detection of oral health issues; facilitates preventive dental care; reduces plaque and gingival indices	Preventive dentistry	AI for self-monitoring; smartphone-based diagnostic support; early prevention strategies
[26] Fu Y et al.	This systematic review evaluates smartphone-based hand function assessment tools that leverage artificial intelligence. It focuses on how sensor data from mobile devices can objectively measure hand mobility and performance, potentially improving rehabilitation and clinical assessment. The study highlights the strengths of combining smartphone sensors with automated analysis while noting gaps in standardization and clinical validation.	Smartphone-based assessment of hand function using AI and sensor data.	Provides objective measurement of hand function; enhances rehabilitation assessment; supports remote monitoring	Hand function assessment	Sensor data integration; AI-driven functional evaluation; clinical assessment support
[27] Negi S et al.	This umbrella review synthesizes evidence on artificial intelligence applications for dental caries diagnosis and detection. It explores a range of models and techniques used to identify caries from dental images and clinical data, summarizing diagnostic performance and limitations. The review underscores the promise of AI in improving diagnostic accuracy while calling for further validation across diverse populations.	AI for dental caries diagnosis and detection across imaging and clinical datasets.	Improves diagnostic accuracy; supports early detection workflows; highlights need for broader validation	Dental caries diagnosis	Diagnostic AI models; image and data fusion techniques; clinical applicability and validation needs
[28] Lange M et al.	This review identifies approaches for using AI in workplace health promotion and prevention, focusing on how digital tools can support occupational wellbeing. It synthesizes evidence on predictive models and interventions that address physical activity, stress management, and healthy behaviours. The study highlights opportunities and challenges for implementing AI-driven workplace health solutions across diverse occupational settings.	AI in workplace health promotion and preventive interventions.	Supports health behaviour monitoring; predictive models for stress and activity; enhances occupational health strategies	Workplace health promotion	AI for behavioural health support; digital intervention trends; organizational implementation
[29] Chow SM et al.	This systematic review and meta-analysis examines the effectiveness of mHealth apps on adherence to and symptoms from oral anticancer medications. It evaluates how mobile applications influence medication adherence, symptom reporting, and clinical outcomes. Results indicate that mHealth interventions can improve adherence and reduce symptom burden, although study heterogeneity suggests the need for standardized outcomes.	Effectiveness of mHealth apps on adherence and symptoms for oral anticancer medications.	Improves medication adherence; reduces symptom burden; supports patient engagement	Oral anticancer treatment adherence	mHealth for adherence support; symptom tracking; patient engagement trends
[30] Jin K et al.	This systematic review examines how smartphone technology integrated with artificial intelligence is used in advanced ophthalmic care. It synthesizes evidence from 52 studies on screening, disease detection, telemedicine, and patient management across major ocular conditions. The review emphasizes improvements in diagnostic performance and accessibility, while noting challenges related to data privacy, algorithm validation, and real-world implementation.	Integration of smartphone technology and AI to improve ophthalmic diagnosis, screening, telemedicine, and patient care.	Enhances early detection and monitoring of eye diseases; supports remote patient management; improves accessibility in underserved areas	Ophthalmic care and eye disease management	AI-driven smartphone imaging; tele-ophthalmology enhancement; accessibility and diagnostic performance improvements
[31] Choi A et al.	This review assesses digital phenotyping methods using smartphone sensors to detect behavioral patterns associated with stress, anxiety, and mild depression in nonclinical adult populations. It highlights evidence that passive sensing, such as GPS, accelerometer, and phone usage patterns, can identify correlates of mild mental health symptoms. The study also addresses challenges such as sensor accuracy, privacy concerns, and individual behavioral variability across contexts.	Digital phenotyping via smartphone sensors for detecting stress, anxiety, and mild depression.	Identifies behavioral patterns linked to mental health using smartphone data; highlights effective passive sensors; discusses ethical and technical challenges	Behavioral mental health monitoring	Passive sensing for behavioral markers; sensor pattern associations with mental health; privacy and technical limitations
[32] Segur-Ferrer J et al.	This scoping review and thematic analysis identifies methodological frameworks and key dimensions to be considered in digital health technology assessment (DHTA). It synthesizes frameworks used to evaluate digital health technologies, including metrics related to clinical effectiveness, usability, economic impact, and ethical considerations. The review highlights the need for comprehensive, multidimensional assessment approaches to support robust evaluation and regulatory decisions.	Frameworks and dimensions for assessing digital health technologies, including AI and mobile health tools.	Clarifies assessment criteria for digital health technologies; highlights clinical, usability, economic, and ethical dimensions; guides structured evaluation practices	Digital health technology assessment	Multidimensional evaluation criteria; methodological framework synthesis; implications for regulatory and policy decisions
[33] Giunti G et al.	This review explores the co-creation of an automated mHealth apps systematic review process using generative AI within a design science research framework. It discusses how AI-enabled tools can assist in automating literature screening, data extraction, and synthesis tasks. The study highlights the potential of generative AI to enhance efficiency and consistency in systematic review workflows while considering design challenges in human–AI collaboration.	Use of generative AI to automate components of systematic review processes for mHealth apps.	Shows generative AI supporting automation of review screening and extraction; enhances efficiency and consistency of review workflows; discusses human–AI cooperation design	Review process automation	Generative AI for review automation; human–AI collaboration challenges; efficiency improvements in evidence synthesis
[34] Pala D et al.	This review examines smartphone applications designed to support nutrition, focusing on target outcomes and core functionalities. It synthesizes evidence on app features such as dietary tracking, personalized feedback, and behavior change support. The study discusses how apps influence nutritional outcomes and user engagement, as well as limitations related to evidence quality and long-term adherence.	Functionality and outcomes of nutrition support smartphone applications.	Offers dietary tracking and personalized feedback; supports behavior change and nutritional monitoring; highlights engagement features	Nutrition support via mobile apps	Personalized nutrition feedback; behavior change support; functionality outcome mapping
[35] Jahan E et al.	This systematic review and meta-analysis evaluates smartphone applications aimed at preventing type 2 diabetes through lifestyle change support. It assesses outcomes related to weight management, physical activity, dietary behaviors, and metabolic indicators. The findings suggest that mHealth apps can contribute to diabetes prevention through structured interventions and continuous user engagement.	Smartphone apps for prevention of type 2 diabetes through lifestyle modification.	Promotes physical activity and dietary change; facilitates continuous engagement; supports metabolic outcome improvements	Type 2 diabetes prevention	Lifestyle modification support; engagement and adherence trends; preventive health impact
[36] Gheisari M et al.	This multidimensional systematic review examines mobile apps for COVID-19 detection and diagnosis, focusing on their potential use in pandemic control and future outbreak management. It synthesizes evidence on app functionalities, diagnostic performance, and integration with public health monitoring systems. The review highlights opportunities and limitations in using mobile tools for pandemic preparedness and response.	Mobile apps for COVID-19 detection and diagnosis and implications for future pandemic control.	Supports disease surveillance and early detection; integrates diagnostic functions with public health data; highlights limitations and future needs	Pandemic detection and control	Diagnostic surveillance via apps; pandemic preparedness relevance; integration with public health infrastructure
[37] Tomlin HR et al.	This review identifies challenges and opportunities for professional medical publication writers to contribute to plain language summaries (PLS) within AI/ML research environments. It discusses barriers related to interpretability, accuracy, and audience comprehension, and highlights strategies to improve the quality of PLS. The study emphasizes the role of clear communication in translating complex AI/ML evidence for diverse stakeholders.	Challenges and opportunities in creating plain language summaries in an AI/ML research context.	Highlights interpretability and communication barriers; suggests strategies for improving PLS quality; addresses stakeholder engagement in evidence translation	Health communication and AI/ML interpretation	Plain language science communication; stakeholder comprehension trends; role of writers in AI evidence translation
[38] Xue J et al.	This scoping review evaluates the current state of chatbots in digital health, examining their functionalities, applications, and potential benefits. It synthesizes evidence on how conversational agents support health information, triage, patient engagement, and self-management. The study discusses both opportunities and limitations, including accuracy, user trust, and integration with clinical workflows.	Evaluation of chatbot technologies in digital health contexts.	Supports health information delivery and triage; enables patient engagement and self-management; discusses challenges with trust and clinical integration	Digital health chatbots	Conversational AI applications; engagement and triage support; trust and workflow integration
[39] He X et al.	This scoping review identifies existing barriers faced by direct-to-consumer AI healthcare apps and provides recommendations for future design improvements. It highlights issues such as usability, transparency, data privacy, and regulatory compliance that limit adoption. The review emphasizes the need to address these challenges to enhance user trust and app effectiveness in everyday healthcare.	Barriers and design recommendations for direct-to-consumer AI healthcare applications.	Identifies usability and transparency challenges; highlights privacy and regulatory issues; offers design recommendations for future apps	Direct-to-consumer AI healthcare apps	Usability and trust barriers; privacy and regulatory considerations; future design directions
[40] Sumner J et al.	This systematic review evaluates AI applications in physical rehabilitation, examining availability, clinical effects, and implementation barriers. Evidence spans app-based systems, robotic devices, gaming systems, and wearables used to restore function, improve physical activity, or assist recovery. The review highlights mixed clinical effects and identifies challenges such as technology literacy, reliability issues, and user fatigue, while noting potential improvements in access and remote monitoring.	AI applications in physical rehabilitation to support recovery and functional outcomes.	AI-enhanced systems for rehabilitation tasks; supports remote monitoring and access to programmes; reduces manpower requirements	Physical rehabilitation	Diverse AI solutions (robotics, wearables); mixed clinical effectiveness; implementation and usability barriers
[41] Bendotti et al.	This systematic review and meta-analysis assesses conversational AI interventions for smoking cessation, focusing on their effectiveness compared with standard care or no intervention. It synthesizes evidence from RCTs using chatbots and dialogue systems across apps, social media, and web platforms. Results indicate promising benefits for sustained tobacco abstinence, though high heterogeneity and study bias suggest cautious interpretation.	Conversational AI for supporting smoking cessation outcomes.	Conversational AI increases likelihood of quitting smoking; chatbots emulate personalised support; highlights engagement and behavioural support	Smoking cessation support	AI for behaviour change support; mobile conversational agents; engagement and intervention heterogeneity
[42] Pyper et al.	This scoping review examines digital health technologies using patient-generated data for real-world clinical outcome measurement. It highlights frameworks and use cases where mobile tools complement clinical assessments, addressing challenges like data quality, integration, and interpretability. The review underscores potential for personalised healthcare decision-making.	Digital health technologies leveraging patient-generated data for clinical outcome measurement.	Uses mobile and digital tools to capture real-world health data; supports objective outcome measurement; highlights integration and interpretability challenges	Patient-generated outcome measurement	Real-world data integration; mobile data for outcomes; challenges in data quality and clinical integration
[43] Hasan et al.	This systematic review and meta-analysis evaluates smartphone-based AI systems for diabetic retinopathy detection. Evidence on sensitivity, specificity, and diagnostic performance shows potential for early identification, especially in low-resource settings. Limitations include data diversity, algorithm generalisability, and need for rigorous clinical validation.	Diagnostic accuracy of smartphone-based AI systems for diabetic retinopathy detection.	AI improves early detection accuracy; facilitates screening in non-specialist settings; highlights validation and generalisability needs	Diabetic retinopathy diagnosis	Smartphone AI for imaging diagnosis; accessibility in low-resource settings; need for broader validation
[44] Chatterjee et al.	This systematic review and knowledge mapping explores ICT-based remote and automatic COVID-19 patient monitoring and care. It highlights mobile apps, telemonitoring systems, and automated alerts used for tracking symptoms, vital signs, and care coordination. The review discusses opportunities and limitations, including data integration, user acceptance, and scalability.	ICT-based remote and automatic monitoring and care for COVID-19 patients.	Mobile and digital tools for remote symptom tracking; supports care coordination and alerting; highlights scalability and integration challenges	COVID-19 remote patient monitoring	Remote monitoring trend; ICT and mobile integration; pandemic-driven innovation
[45] Fegan & Hutchinson	This short review examines the potential of mobile technology to address early childhood caries. It discusses current interventions, smartphone-enabled dental monitoring, and preventive strategies. The article emphasizes the need for stronger evidence and standardized methodologies for real-world prevention.	Exploration of mobile technology’s role in reducing early childhood caries.	Suggests mobile tools for dental health monitoring; encourages preventive strategies; notes need for rigorous validation	Early childhood caries prevention	Mobile dental monitoring concepts; preventive focus; need for stronger evidence
[46] Koumpouros et al.	This systematic review investigates mobile applications for pain management, covering commercial and research efforts integrating AI or computational support. It synthesizes evidence on app features, usability, and outcomes related to tracking pain, adherence, and self-help resources. Limitations include diversity of approaches, standardization, and clinical validation.	Pain management mobile applications integrating AI and supportive technologies.	Supports pain tracking and self-management; offers therapeutic guidance and reminders; highlights usability and evidence gaps	Pain management via mobile apps	Self-management trend; pain tracking features; usability and validation limitations
[47] Duarte et al.	This systematic review evaluates digital clinical tools for screening or diagnosing obstructive sleep apnea. It synthesizes evidence on tool performance, including sensitivity, specificity, and feasibility across care settings. The review highlights opportunities for earlier detection and streamlined diagnostics, along with challenges in data quality, patient adherence, and integration.	Digital clinical tools and AI in screening and diagnosis of obstructive sleep apnea.	Enhances early detection of sleep apnea; supports diagnostic workflows; notes challenges in real-world integration	Obstructive sleep apnea screening and diagnosis	AI clinical tool trend; diagnostic accuracy focus; adoption challenges
[48] Lin et al.	This systematic assessment examines conversational agents for mental health and well-being, focusing on scope, characteristics, behaviour change techniques, and app quality. It highlights how these apps use dialogue and interaction patterns to support mental health outcomes, including cognitive behavioural strategies. Limitations in sustaining long-term mental health improvements are also discussed.	Conversational agents for mental health and well-being, including behaviour change and engagement.	Supports mental health engagement via chatbots; integrates behaviour change techniques; evaluates quality and engagement metrics	Conversational mental health support	Behaviour change focus; conversational AI engagement; quality variability
[49] Moharrami et al.	This systematic review evaluates AI algorithms for detecting dental caries on oral photographs, assessing diagnostic accuracy, methodology, and data sources. Evidence shows promising performance for screening and early detection, though limitations include dataset bias and the need for validation across diverse populations.	AI for detecting dental caries from oral photographs and imaging data.	AI enhances dental caries detection accuracy; supports early screening and decision support; highlights dataset and validation needs	Dental caries detection via photography	Imaging AI for dentistry; diagnostic screening trend; validation and bias issues
[50] Piendel et al.	This review provides an update on mobile applications that collect data among adults with or at risk of Alzheimer’s disease, focusing on passive and active data collection relevant to cognitive changes. It synthesizes evidence on how smartphone apps can monitor daily routines and behaviors that may indicate subtle cognitive decline, with potential utility for early detection and screening. The review also discusses limitations in current app validation and privacy issues, noting that widespread implementation remains underdeveloped despite promising feasibility.	Mobile applications collecting data for cognitive health and Alzheimer’s disease screening.	Passively and actively collects cognitive data; potential early detection and screening support; highlights privacy and validation concerns	Cognitive health screening and Alzheimer’s risk	Passive and active data tracking; cognitive change monitoring; screening tool feasibility
[51] Lam et al.	This systematic review examines applications of markerless motion capture (MMC) technology for clinical measurement in rehabilitation, highlighting its potential to track movement without physical markers. It summarizes studies where MMC has been applied to identify and measure movement patterns in various patient populations, with focuses on clinical utility and feasibility. The review notes that although the approach shows promise for rehabilitation assessments, evidence on clinical effectiveness and integration with AI models remains preliminary and requires further development.	Markerless motion capture technology applied to clinical movement measurement in rehabilitation.	Avoids need for physical markers; tracks movement kinematics; potential integration with AI for screening	Rehabilitation movement analysis	MMC for clinical assessment; motion tracking innovations; preliminary evidence and integration needs
[52] Sumra et al.	This systematic review evaluates smartphone apps designed for domestic violence prevention, focusing on features and effectiveness of mobile tools addressing safety, reporting, and support. It synthesizes evidence on how these apps facilitate emergency response, risk assessment, and access to resources for individuals at risk of domestic violence. The study highlights the range of functionalities offered and notes gaps in empirical evaluation and standardized outcome measurement for these prevention technologies.	Smartphone apps for domestic violence prevention and support.	Supports emergency reporting and risk assessment; provides safety resources and guidance; notes evaluation and outcome measure gaps	Domestic violence prevention	Safety and reporting functions; support resource access; need for standardized evaluation
[53] Chakraborty et al.	This systematic review examines the role of telehealth startups in transforming healthcare service delivery, synthesizing evidence on digital platforms, service models, and integration with clinical care. It highlights how telehealth innovations, including mobile technologies, facilitate remote consultations, monitoring, and care coordination, expanding access to healthcare services. The review also discusses barriers such as regulatory issues, interoperability challenges, and digital literacy that affect implementation and uptake.	Telehealth startups and their impact on healthcare service delivery, including digital and mobile integration.	Expands remote consultations and care coordination; integrates monitoring and patient engagement; notes regulatory and interoperability challenges	Telehealth service delivery	Remote care expansion; digital platform integration; implementation barriers
[54] Irgang et al.	This systematic review explores data-driven technologies used to prevent surgical site infections by enabling early detection, risk stratification, and monitoring. It synthesizes approaches that leverage AI, predictive analytics, and mobile or clinical data platforms to support infection prevention workflows. The study highlights technological contributions to value creation in healthcare by improving surveillance, informing clinical decisions, and guiding intervention strategies.	Data-driven technologies, including AI, for preventing surgical site infections.	Enhances infection surveillance and prediction; supports clinical decision-making; guides prevention strategies	Surgical site infection prevention	Predictive analytics for infection risk; AI surveillance approaches; value creation in prevention
[55] Torres-Guzman et al.	This systematic review examines the effectiveness of smartphones and threshold-based monitoring methods for remote fall detection, focusing on accuracy and feasibility in real-world settings. It synthesizes evidence showing that these methods can reliably detect falls using sensor data and predefined thresholds, with implications for timely response and patient safety. The review also discusses variability in algorithms, sensor performance, and usability factors that influence effectiveness in diverse populations.	Smartphone and threshold-based monitoring for remote fall detection.	Detects falls remotely using sensor thresholds; improves timeliness of alerts; highlights algorithmic and usability variability	Remote fall detection	Sensor-based monitoring; remote safety support; algorithm and usability considerations
[56] van Eijck et al.	This scoping review examines digital health applications designed to establish remote diagnosis of orthopedic knee disorders, focusing on mobile and digital tools that assess symptoms, function, and imaging. It synthesizes evidence on tele-assessment, diagnostic decision support, and remote clinical evaluation methods that leverage AI or automated analysis. The study highlights potential benefits in reducing in-person visits while noting challenges in validation, clinical adoption, and integration into care pathways.	Remote digital health applications for diagnosis of orthopedic knee disorders.	Enables remote clinical symptom and function assessment; supports diagnostic workflows; notes validation and adoption challenges	Orthopedic knee disorder diagnosis	Remote assessment trends; digital diagnostic tools; integration challenges
[57] Rahman et al.	This research review and content analysis evaluates mobile apps aimed at preventing violence against women and girls (VAWG), assessing features, reach, and content quality. It synthesizes evidence on app functionalities such as risk assessment, safety planning, incident reporting, and educational resources. The study highlights gaps in empirical validation and standard outcome measures, calling for improved evaluation frameworks and user-centered design.	Mobile apps for preventing violence against women and girls, focusing on safety and support features.	Provides risk assessment and safety planning tools; supports incident reporting and education; highlights evaluation and design gaps	Violence against women and girls prevention	Safety and education features; support tool functionalities; need for robust evaluation
[58] Abdul Latif El Ejel et al.	This systematic review examines digital diabetes management technologies for type 2 diabetes care in home-based settings, focusing on mobile tools that support self-management, monitoring, and behaviour modification. It synthesizes evidence on features such as glucose tracking, personalised feedback, and integration with clinical workflows that aim to improve glycemic control and patient engagement. The review discusses benefits and limitations, noting variability in evidence quality and the need for long-term effectiveness studies.	Home-based digital technologies for managing type 2 diabetes.	Facilitates glucose and lifestyle monitoring; provides personalised feedback; enhances patient engagement	Type 2 diabetes home-based management	Self-management support; personalised monitoring; evidence variability and outcome gaps
[59] Jafari et al.	This systematic review examines the effect of home-based and remote exercises on low back pain during the COVID-19 pandemic, synthesizing evidence on pain reduction, functional improvement, and participation outcomes. It reviews mobile and remote exercise interventions that blend digital guidance, monitoring, and user engagement. The study highlights positive effects on pain and function, while noting heterogeneity in intervention designs and outcome measures.	Effects of home-based and remote exercise interventions for low back pain.	Supports pain reduction and functional improvement; utilises mobile guidance and engagement; notes intervention design variability	Low back pain management	Remote exercise benefits; mobile engagement features; heterogeneous outcomes
[60] Vijendran et al.	This systematic review evaluates the effectiveness of smartphone technology for detecting pediatric ocular diseases. It summarizes diagnostic accuracy, feasibility, and applicability of mobile-based imaging and AI-assisted analysis in clinical and remote settings. The review also discusses limitations related to validation, accessibility, and integration with existing ophthalmic care pathways.	Smartphone-based detection of pediatric eye diseases.	Enables early detection using mobile imaging; supports AI-assisted diagnostics; facilitates remote and clinical screening	Pediatric ophthalmology	Mobile diagnostic tools; AI-assisted evaluation; early detection potential
[61] Andrade & Viñán-Ludeña	This comprehensive review maps research on ICT addiction, covering internet, smartphone, social media, and gaming addictions. It synthesizes evidence on assessment methods, prevalence, risk factors, and interventions, highlighting trends in measurement and prevention. The review also notes gaps in standardization and longitudinal studies to assess long-term outcomes.	ICT addiction, including internet, smartphone, social media, and gaming.	Summarizes assessment and intervention strategies; identifies prevalence and risk factors; highlights research gaps in standardization	Behavioral and mental health	Technology use patterns; risk and prevalence mapping; intervention development
[62] Sylla et al.	This rapid systematic review examines 25 years of digital health progress in low- and middle-income countries toward universal health coverage. It highlights the role of mobile and AI-enabled tools in improving access, quality, and efficiency of healthcare delivery. The review discusses implementation challenges, equity issues, and lessons learned from large-scale digital health initiatives.	Digital health in LMICs for universal health coverage.	Enhances access and healthcare efficiency; supports AI and mobile interventions; identifies barriers to scaling and equity	Global health/LMICs	Digital health adoption trends; AI and mobile integration; coverage and equity challenges
[63] Schaap et al.	This scoping review assesses the suitability of just-in-time adaptive interventions (JITAIs) for post-COVID-19-related symptoms. It synthesizes evidence on mobile and AI-enabled interventions delivering tailored, real-time support to patients. The review notes effectiveness in symptom monitoring and personalized feedback, while also highlighting design, engagement, and evaluation challenges.	Just-in-time adaptive interventions for post-COVID-19 symptoms.	Provides real-time adaptive support; monitors symptoms and offers feedback; uses mobile and AI tools for personalization	Post-COVID symptom management	Personalized intervention delivery; mobile and AI application; engagement and feasibility issues
[64] Zhuang et al.	This systematic review and meta-analysis evaluates digital health interventions for chronic obstructive pulmonary disease (COPD). It synthesizes evidence on mobile apps, remote monitoring, and AI-driven management tools, assessing impact on clinical outcomes and patient adherence. The review highlights benefits in self-management and symptom tracking, while noting variability in intervention designs and evidence quality.	Digital health interventions for COPD management.	Supports symptom monitoring and self-management; AI aids in risk prediction and adherence; facilitates remote patient care	COPD	Remote monitoring and management; AI-assisted decision support; intervention variability
[65] Zhong et al.	This systematic review explores the use of digital phenotyping to differentiate unipolar depression from bipolar disorder. It examines smartphone and wearable data for behavioral, cognitive, and physiological markers that may improve diagnostic precision. The review discusses the potential for personalized assessment and early intervention, along with limitations in validation and standardization.	Digital phenotyping for mood disorder differentiation.	Enables behavioral and physiological monitoring; supports diagnostic precision; guides personalized mental health assessment	Mental health/Mood disorders	Digital phenotyping trends; AI-assisted differentiation; early intervention potential
[66] Kargarandehkordi et al.	This systematic review investigates the integration of wearable biosensors with AI for mental health monitoring. It summarizes applications in detecting stress, anxiety, and mood disorders through physiological and behavioral data. The review highlights opportunities for continuous monitoring and personalized interventions, while noting challenges in data quality, algorithm validation, and clinical adoption.	Wearable biosensors combined with AI for mental health monitoring.	Provides continuous physiological and behavioral monitoring; supports early detection of mental health conditions; enables personalized interventions	Mental health/Wearables	Wearable-AI integration; continuous monitoring; early detection and personalization
[67] Melia et al.	This review examines applications of AI to ecological momentary assessment (EMA) data in suicide research. It synthesizes methods for predicting suicidal ideation and behavior using real-time digital and mobile data. The review emphasizes opportunities for early risk detection and intervention planning, alongside ethical, privacy, and methodological challenges.	AI applied to EMA data for suicide research.	Predicts suicidal ideation and behavior; supports real-time monitoring and intervention; highlights ethical and methodological considerations	Suicide prevention/Mental health	EMA-based prediction; AI-assisted risk assessment; privacy and ethics concerns
[68] Woll et al.	This systematic review evaluates AI applications in linking device-based physical activity assessment with mental health outcomes. It synthesizes evidence on machine learning approaches to analyze activity patterns and predict psychological well-being. The review notes potential for personalized interventions, monitoring, and mental health promotion, as well as challenges in study design and data integration.	AI and device-based physical activity assessment for mental health.	Correlates activity data with mental health outcomes; supports personalized intervention design; utilizes AI for behavioral pattern analysis	Physical activity and mental health	Activity-mental health correlations; AI-assisted monitoring; personalized intervention insights
[69] Dehbozorgi et al.	This systematic review investigates AI applications in mental health, covering diagnosis, treatment, monitoring, and prediction. It synthesizes evidence from diverse tools including mobile apps, wearables, and predictive algorithms, highlighting clinical and methodological contributions. The review also discusses limitations in implementation, validation, and scalability, emphasizing the need for robust evidence before clinical deployment.	AI applications in mental health care.	Supports diagnosis, monitoring, and treatment; utilizes predictive algorithms and mobile tools; highlights clinical and methodological contributions	Mental health care	AI-enabled interventions; remote monitoring; evidence gaps and implementation challenges
[70] van Genugten et al.	This systematic review qualitatively examines the current state of just-in-time adaptive interventions (JITAIs) in mental health. It evaluates design, implementation, and effectiveness, highlighting challenges and opportunities for personalized digital interventions. The review also identifies areas for methodological improvements and future research directions.	Just-in-time adaptive interventions for mental health.	Supports real-time, personalized mental health interventions; highlights design and implementation strategies; provides guidance for future development	Mental health	JITAI design trends; implementation challenges; personalized intervention approaches
[71] Abdulazeem et al.	This bibliometric analysis and report reviews the use of digital health technologies for dementia care. It summarizes research trends, intervention types, and adoption patterns, emphasizing AI and mobile solutions for monitoring, support, and caregiver assistance. The review also identifies gaps in accessibility, evaluation, and long-term outcomes.	Digital health technologies in dementia care.	Supports remote monitoring and caregiver support; highlights AI-enabled dementia interventions; maps research trends and adoption patterns	Dementia care	Technology adoption trends; AI applications; research and evaluation gaps
[72] Pan et al.	This bibliometric analysis examines the application of artificial intelligence in chronic disease management. It evaluates trends in AI tools for risk prediction, monitoring, and personalized interventions. The review also discusses adoption barriers, research hotspots, and integration with digital health platforms.	AI for chronic disease health management.	Enables monitoring and risk prediction; supports personalized care; highlights adoption and research trends	Chronic disease management	AI-assisted monitoring; personalized intervention applications; research hotspots and trends
[73] Genovese et al.	This systematic review investigates AI applications in clinical language translation and interpretation. It evaluates performance, usability, and integration of AI tools to support patient-provider communication. The review also identifies challenges in accuracy, clinical adoption, and cross-cultural considerations.	AI for language translation and interpretation in clinical settings.	Facilitates patient-provider communication; improves accessibility for multilingual populations; supports clinical decision-making	Clinical communication	AI-assisted translation tools; multilingual clinical applications; accuracy and adoption challenges
[74] Akbarian et al.	This systematic review examines features and technologies used in mobile health applications for inflammatory bowel disease. It synthesizes evidence on symptom tracking, monitoring, and patient self-management functionalities. The review also highlights gaps in integration, usability, and personalization of interventions.	mHealth apps for inflammatory bowel disease management.	Supports symptom monitoring and self-management; facilitates patient engagement; integrates mobile and AI functionalities	Inflammatory bowel disease	App feature trends; self-management support; gaps in personalization and usability
[75] Bernard et al.	This systematic review evaluates self-management support apps for individuals with spinal cord injury. It analyzes app functionalities, usability, and evidence for improving patient outcomes. The review also identifies barriers to adoption and potential enhancements for accessibility and engagement.	Self-management support apps for spinal cord injury.	Facilitates remote self-management and monitoring; provides educational and motivational support; highlights usability and accessibility issues	Spinal cord injury	App functionality trends; remote self-management; usability and engagement considerations
[76] Gao et al.	This meta-analysis investigates AI-based facial recognition for detecting obstructive sleep apnea. It synthesizes diagnostic accuracy, validation methods, and clinical applicability of AI algorithms. The review highlights opportunities for non-invasive, rapid screening while noting limitations in generalizability and standardization.	AI facial recognition for obstructive sleep apnea detection.	Enables non-invasive screening; supports AI-assisted diagnosis; provides rapid risk assessment	Sleep disorders/Obstructive sleep apnea	AI diagnostic trends; non-invasive screening; standardization challenges

**Table 4:** Main macro-areas identified in AI-driven mHealth research.

Macro Area	Included Studies	Evidence Maturity Level	Typical Interventions/Focus	Notes/Research Gaps	Key Studies [ ]
Mental Health & Well-being	12	Developing	AI for predicting mental health symptoms, stress, mood disorders; conversational agents; Just-in-Time adaptive interventions	Emerging interest in personalized, proactive interventions; need more real-world evaluation	[21,31,41,48,61,63,65,66,67,68,69,70]
Disease Prevention & Management	5	Developing	mHealth apps for chronic disease prevention (diabetes, domestic violence, rehabilitation, Alzheimer’s monitoring)	Evidence fragmented; heterogeneous outcomes; limited AI integration	[22,35,40,50,52]
AI & Mobile Health Technologies	5	Emerging	Frameworks for AI-enabled mHealth app development, assessment of chatbots and COVID-19 detection apps	Methodological guidance still underdeveloped; limited empirical evaluation	[32,33,36,38,39]
Cancer & Oral Health	5	Developing	AI for oral cancer detection, dental caries, self-monitoring via smartphone images	Mostly early-stage studies; few large-scale validations	[23,25,27,45,49]
Chronic Diseases & Diagnostics	11	Developing	AI in diabetic retinopathy, sleep apnea, COPD, fall detection, pediatric eye screening	Need standardized datasets; cross-platform integration; longitudinal studies	[30,43,47,54,55,58,60,64,72,74,76]
Health Data & Outcome Measurement	4	Emerging	Patient-generated data for real-world outcomes, cognitive support apps	Limited data on long-term impact; interoperability challenges	[38,42,50,71]
Workplace Health	1	Emerging	AI for health promotion and prevention in occupational settings	Few studies; more evidence needed across diverse workplaces	[28]
Health Technologies & Innovations	5	Developing	Remote rehabilitation, markerless motion capture, pain management, spinal cord injury apps	Evidence growing but heterogeneous; standardization lacking	[24,46,51,59,75]
Healthcare Technology Integration	4	Emerging	Telehealth, remote monitoring, AI translation tools	Integration into clinical workflows underexplored	[44,53,56,73]
Technology for Medication Adherence	1	Emerging	mHealth apps for oral anticancer therapy adherence	Limited to specific conditions; broader applicability unclear	[29]
Nutrition & Health	1	Emerging	Apps for nutritional support and dietary management	Evidence sparse; outcomes not standardized	[34]
Public Health	3	Emerging	mHealth for violence prevention, low-income country coverage	Early-stage implementation; scaling challenges	[52,57,62]

**Table 5:** Emerging opportunities and areas needing a broader investigation.

Study	Opportunities	Areas Needing Broader Investigation
[21]	AI and mHealth platforms present valuable opportunities for predicting and managing mental health conditions such as anxiety, stress, and depression, particularly in youth. Real-time monitoring can provide timely interventions, improving mental well-being.	Privacy concerns are significant, as mental health data is highly sensitive. Furthermore, accessibility remains a challenge, especially in marginalized communities with limited access to mobile technologies.
[22]	AI can facilitate the early detection, diagnosis, and management of neglected tropical diseases (NTDs), particularly in resource-limited settings. Mobile apps help expand reach, improving pandemic preparedness and surveillance.	Low-tech infrastructure in many parts of the world can hinder the implementation of AI-powered health solutions. Furthermore, integrating these technologies into already strained healthcare systems remains a considerable challenge.
[23]	AI applications in oral health can enable early diagnosis of oral cancer and improve the detection of dental conditions such as caries. Mobile platforms can empower individuals to self-monitor, enhancing preventive care.	Despite the promise, there is resistance to integrating AI into traditional dental practices, and limited data hinders the development of robust AI tools. Additionally, costs may be prohibitive for some dental practices.
[24]	AI-powered mobile apps for patient monitoring can track chronic conditions like diabetes and hypertension, allowing for more personalized and proactive care. These tools provide valuable insights for both patients and healthcare providers.	Security and privacy concerns are a major challenge when handling sensitive health data. Inaccuracies in symptom tracking can also result in improper interventions or unnecessary healthcare costs.
[25]	AI in dental care can improve the diagnosis of dental diseases and promote preventive care. Mobile technologies enable continuous monitoring, fostering a more patient-centered and proactive approach to oral health management.	Patient engagement with AI tools in dental health is limited, and skepticism remains around their effectiveness. Additionally, regulatory hurdles exist in ensuring these tools meet medical standards.
[26]	AI offers substantial promise for improving diagnostic accuracy and streamlining healthcare practices, particularly for chronic disease management. Mobile health technologies also promote self-monitoring and timely intervention.	Over-reliance on AI systems in diagnostics can be problematic, particularly if clinical oversight is inadequate. Furthermore, integrating AI into healthcare systems without disrupting existing practices remains a significant challenge.
[27]	AI can improve the early diagnosis of oral health issues, by analyzing oral photographs and other diagnostic tools. This could significantly enhance early-stage detection and patient outcomes.	Challenges include insufficient validation data, especially regarding long-term accuracy, and resistance to adopting AI-based tools within traditional dental settings.
[28]	AI can assist in chronic disease management by providing personalized treatment plans and continuously monitoring health metrics. AI supports individualized health programs, while mHealth platforms extend interventions to underserved groups.	Data accuracy is crucial for effective AI-powered interventions, and patient adherence to these tools can be inconsistent. Furthermore, issues related to regulatory approval and integration into clinical practice remain. Limited evidence on AI’s broader applicability and concerns over data privacy and ethical implementation.
[29]	AI-based mobile health applications enable real-time symptom tracking and personalized health management, especially for chronic conditions. They can also enhance medication adherence and reduce hospital visits as in the case in anticancer oral medications.	User data privacy remains a significant concern, as sensitive health information is collected. There is also a need for further research to demonstrate the clinical efficacy of these AI-driven mobile apps.
[30]	AI in mobile health is revolutionizing patient monitoring and health management, especially for chronic diseases (including ophthalmology). It allows for continuous feedback, enabling more precise interventions and improving patient outcomes.	Potential challenges include insufficient patient engagement, inaccuracies in symptom tracking, and privacy concerns regarding the handling of sensitive health data.
[31]	AI can provide real-time interventions for mental health, offering tailored strategies for managing stress and promoting overall well-being. The focus on youth mental health is particularly promising.	Privacy and security concerns are prominent, as mental health data is especially sensitive. Additionally, achieving equitable access across diverse populations remains a challenge.
[32]	AI has the potential to enhance telehealth services, improving patient access and streamlining healthcare delivery. AI-powered systems can also enhance remote consultations, making healthcare more efficient.	Barriers include regulatory challenges, particularly with regard to cross-border healthcare delivery. There are also concerns about trust and reliance on AI systems for critical healthcare decisions.
[33]	The integration of AI into telehealth could transform healthcare systems, improving efficiency, access, and patient outcomes. Generative AI models automate the extraction, classification, and synthesis of data from mHealth app reviews. AI can assist in creating structured summaries, identifying trends, and highlighting key functionalities across various mHealth apps.	Ethical concerns about AI’s decision-making in healthcare, accountability, and the potential for biased systems need to be addressed. Legal frameworks for AI in healthcare are also still developing. Ensuring the AI tools are accurate and unbiased, and managing the complexity of app diversity and rapid development in the mHealth sector.
[34]	AI applications in chronic disease prevention and management can lead to personalized health plans, promoting healthier lifestyles and preventing diseases like diabetes and cardiovascular conditions.	Achieving consistent patient engagement and overcoming data privacy concerns are key obstacles. Additionally, ensuring regulatory compliance for AI applications in healthcare is an ongoing challenge.
[35]	AI-powered apps provide opportunities for preventing and managing chronic diseases by offering personalized recommendations, including lifestyle and dietary interventions, ultimately reducing healthcare costs.	Integration of AI into clinical workflows remains a challenge, as healthcare professionals may be hesitant to adopt AI tools. Moreover, ensuring these apps are both scientifically valid and effective is crucial for widespread acceptance.
[36]	AI-driven mobile apps provide new ways to manage infectious diseases through early detection, real-time monitoring, and surveillance. This has proven particularly valuable in the context of pandemics, such as COVID-19.	Variability in data quality across regions poses a challenge for AI’s effectiveness in disease management. Moreover, patient trust in AI-driven tools during health crises can be difficult to establish.
[37]	AI integration, including in telehealth platforms, has the potential to improve efficiency, reduce administrative costs, and enhance patient care. Real-time data processing can lead to more accurate diagnoses and treatments. AI-based simplification tools can enhance the accessibility of health information for patients and caregivers.	Resistance from healthcare workers and patients to AI-powered healthcare systems is a significant barrier, and ensuring these systems are secure and trustworthy is crucial.
[38]	AI can play a pivotal role in personalized treatment and monitoring of chronic conditions, leading to better management and reduced healthcare costs. Continuous monitoring through mobile health tools can facilitate early interventions. Chatbots are a promising tool for delivering accessible and personalized health interventions.	Data security and privacy concerns remain one of the primary obstacles. Additionally, AI systems must be properly calibrated to avoid misdiagnoses or ineffective interventions.
[39]	AI holds immense potential in health monitoring, offering tools for detecting and managing health conditions. DTC AI apps provide consumers with the potential for real-time health monitoring and diagnosis, improving health management.	Ethical issues related to AI decision-making, require careful consideration. Risks include data privacy issues, the accuracy of AI-driven diagnoses, and the need to ensure users’ trust in these technologies.
[40]	AI-driven rehabilitation technologies, such as markerless motion capture, enable more personalized and efficient physical therapy. These tools have the potential to significantly improve rehabilitation outcomes.	Resistance to new technologies, especially in the rehabilitation field, can delay adoption. Furthermore, clinical validation and integration into existing rehabilitation practices are necessary for success.
[41]	mHealth technologies, such as apps and social media platforms, integrated with AI to deliver interventions for tobacco cessation have potential. Conversational AI interventions hold promise for increasing smoking cessation rates in large populations.	Clinician trust in AI tools remains a barrier, as there may be concerns over their reliability in critical healthcare settings. High heterogeneity across studies, varying methodologies, and high participant dropout rates. More consistent and standardized trials are needed.
[42]	AI technologies in mobile health provide continuous patient monitoring, leading to better management of chronic conditions. These platforms offer real-time interventions and personalized care, improving long-term health outcomes. Real-world clinical data collected through DHTs can significantly enhance patient care and health monitoring.	Ensuring data privacy and security in mobile health tools is a major concern. Moreover, inconsistent user adherence to these technologies can hinder their effectiveness in long-term care. The need for more rigorous studies to validate the reliability of real-world data and ensure its integration into clinical decision-making.
[43]	Smartphone-based AI tools offer a promising solution for mass screening of diabetic retinopathy in low-resource settings. Mobile technologies promote preventive care by enabling mass screaning.	Further high-quality studies are needed to validate these tools in real-world clinical environments and ensure their effectiveness. Additionally, insufficient data availability limits the effectiveness of AI systems in certain applications.
[44]	AI has the potential to transform infectious disease management by enabling earlier detection, diagnosis, and real-time monitoring through mobile apps. This could significantly improve response times in pandemics.	Challenges include ensuring widespread accessibility of mobile health tools in low-resource settings and maintaining the accuracy of AI systems in diverse epidemiological environments.
[45]	mHealth apps can be an accessible, low-cost solution for improving oral health education and prevention. These apps provide users with valuable resources such as instructional videos, reminders for oral hygiene routines, and tips for maintaining healthy teeth	Ethical concerns around data usage, privacy, and AI’s decision-making processes need to be carefully addressed. Furthermore, ensuring that AI systems do not perpetuate existing biases in healthcare is a major concern.
[46]	AI-driven innovations in healthcare show promise in transforming disease prevention and management, including new applications for cognitive health and innovative pain management solutions.	Regulatory, ethical, and technical hurdles need to be overcome before these innovations can be implemented widely. The integration of these technologies into existing healthcare practices is also a significant challenge.
[47]	These digital tools could offer cost-effective, scalable solutions for OSA screening and diagnosis, particularly in underserved populations. They provide the opportunity for continuous, at-home monitoring, enabling earlier intervention and personalized care.	The tools still face challenges in terms of external validation and generalizability. Many studies have only validated models internally, and there is a need for further comparison with polysomnography across diverse populations and clinical environments. Additionally, technical limitations in sensor accuracy and data analysis may hinder full adoption.
[48]	AI-driven health applications in mental health management can help predict and prevent mental health crises. These apps can support early interventions for individuals at risk.	Privacy concerns are paramount when dealing with mental health data, and patient engagement with digital tools can vary, affecting their long-term impact.
[49]	AI applications in dental care can lead to more precise diagnoses and earlier interventions, improving patient outcomes. Mobile tools support self-care and early detection of dental issues.	The lack of sufficient clinical evidence to support AI’s efficacy in dental practice remains an issue, along with concerns regarding patient privacy and data security.
[50]	Emerging AI technologies in healthcare offer opportunities for advancements in AD. mHealth apps and AI can collect continuous data on cognitive performance and behavioral changes, allowing for passive monitoring and early identification of cognitive decline in individuals at risk of AD.	Regulatory barriers, including the need for thorough clinical validation of AI tools, remain a significant challenge. Moreover, concerns about patient data privacy and ethical decision-making persist.
[51]	AI technologies in physical rehabilitation, such as markerless motion capture, enable personalized therapy and more efficient recovery. These tools can significantly improve patient outcomes.	The adoption of AI in rehabilitation is slow due to a lack of clinician trust in the technology, and the need for comprehensive clinical trials to validate its efficacy remains a challenge.
[52]	mHealth apps provide emergency assistance, awareness, and legal information to potential victims of domestic violence, enabling discreet and accessible support. AI could enhance the functionality of these apps by incorporating features like speech recognition, sentiment analysis, and real-time emergency alerts based on audio cues.	Data security and privacy concerns continue to be significant, and integrating these technologies. The current lack of automation and AI integration in existing apps.
[53]	Telehealth startups are pivotal in offering teleconsultations, telemonitoring, and electronic health records solutions, focusing on mobile health applications and AI-driven personalized care. AI enhances telehealth through personalized care, digital therapeutics, and the development of wearable device technologies, optimizing healthcare delivery.	Ethical concerns about data usage, privacy, and AI’s role in healthcare decisions must be addressed to ensure equitable and fair healthcare delivery. Key challenges include infrastructure limitations, regulatory hurdles, and ensuring sustainable revenue generation models for telehealth startups.
[54]	AI applications in surgical settings can improve diagnostic accuracy and support surgical decision-making. AI and mobile health solutions offer significant opportunities in reducing healthcare costs, improving patient outcomes, and enhancing infection prevention practices.	The challenge of clinician acceptance and ensuring AI systems integrate seamlessly into existing clinical workflows remains a major obstacle. Standardizing data management, integrating DDTs with existing systems, and ensuring patient data security remain major challenges. Additionally, maintaining patient trust is crucial.
[55]	AI-powered mobile health tools enable more effective management of chronic conditions and improve medication adherence. These technologies offer personalized care and are scalable for widespread use. Smartphone-based fall detection systems provide an opportunity to improve elderly care by enabling real-time intervention and reducing healthcare costs.	Inconsistent adherence to digital health tools by patients is a challenge, as is ensuring the accuracy of data collected through these platforms. Adoption is limited due to technological barriers, such as sensor reliability, and reluctance from older adults to embrace new technologies.
[56]	AI can optimize healthcare systems by improving diagnostics and streamlining care processes, ultimately enhancing patient outcomes and reducing costs.	Challenges include the need for standardization in data collection methods, integrating multiple data sources, and improving diagnostic accuracy.
[57]	Mobile apps provide immediate access to resources and support for preventing violence against women, empowering users with education, safety planning, and emergency contacts. They can raise awareness and foster community engagement.	Ensuring widespread accessibility across different regions and demographics, maintaining user privacy and data security, and guaranteeing timely and reliable emergency responses remain significant hurdles. Cultural sensitivities and trust-building are also critical.
[58]	AI-driven digital diabetes management allows personalized treatment plans, continuous glucose monitoring, and lifestyle coaching at home, improving adherence and health outcomes. It enables scalable chronic disease care beyond clinical settings.	Challenges include ensuring accuracy of self-reported and sensor data, sustaining patient engagement over long periods, integrating AI tools with traditional healthcare providers, and addressing disparities in technology access.
[59]	Home-based and remote exercise interventions supported by digital tools offer safe rehabilitation during pandemics or mobility constraints, enabling continuous care, pain management, and improved physical function.	Barriers include technological literacy of patients, variable internet connectivity, lack of personalized supervision, and difficulties in monitoring and motivating patients remotely to ensure adherence and effectiveness.
[60]	AI-powered smartphone applications can enable early and affordable detection of pediatric ocular diseases, improving access in low-resource settings, and potentially reducing preventable vision loss through timely referrals.	Validating AI diagnostic accuracy in diverse populations, training non-specialist users, ensuring follow-up care, and managing ethical issues related to data privacy and consent pose ongoing challenges.
[61]	Digital tools offer potential for monitoring and intervention in ICT addiction (internet, smartphone, gaming), supporting self-regulation, psychoeducation, and behavioral therapy enhancements via AI personalization.	Risks include the paradoxical effect of increasing screen time, managing user engagement, addressing the stigma associated with addiction, and ensuring interventions are evidence-based and tailored to individual needs.
[62]	Digital health technologies can significantly enhance healthcare delivery in low- and middle-income countries, promoting universal health coverage by overcoming geographical and resource barriers with mobile solutions.	Infrastructure deficits, sustainability of programs, socio-cultural adaptation, regulatory challenges, and digital literacy disparities remain substantial obstacles to widespread adoption.
[63]	Just-in-time adaptive interventions (JITAI) can provide personalized, context-aware support to patients recovering from COVID-19, improving symptom management and quality of life through timely, adaptive feedback.	Challenges include ensuring real-time data reliability, protecting patient privacy, maintaining user engagement, and rigorously validating intervention efficacy in diverse populations.
[64]	Digital health interventions enable continuous monitoring and management of COPD symptoms, facilitate remote consultations, and support patient self-management, potentially reducing exacerbations and hospitalizations.	User adherence over time, integration into clinical workflows, interoperability with healthcare systems, and data security concerns must be addressed to maximize effectiveness.
[65]	Digital phenotyping through AI offers non-invasive methods for distinguishing unipolar depression from bipolar disorder, enabling earlier and more accurate diagnosis and personalized treatment plans.	Privacy and ethical concerns about continuous monitoring, data interpretation challenges, risk of misclassification, and the potential for stigmatization require careful management.
[66]	Wearable biosensors fused with AI provide real-time monitoring of mental health states, enabling timely interventions, personalized care, and better understanding of physiological correlates of mental health.	Ensuring sensor accuracy and reliability, maintaining user comfort and compliance, ethical management of sensitive physiological data, and integrating findings into clinical practice are key challenges.
[67]	AI applied to ecological momentary assessment data can improve suicide risk prediction by analyzing real-time mood and behavior patterns, potentially enabling preventive interventions and saving lives.	Managing false positives and negatives, respecting privacy and consent, ensuring patient engagement, and avoiding over-reliance on automated predictions present ongoing difficulties.
[68]	Device-based physical activity assessment combined with AI can tailor interventions to improve mental health by encouraging physical activity and tracking emotional well-being in a personalized manner.	Maintaining motivation and adherence, ensuring accuracy of mood and activity detection, integrating recommendations into everyday life, and protecting user data are significant challenges.
[69]	AI applications in mental health span diagnosis, treatment, and support, enhancing clinical decision-making, improving access to care, and enabling personalized interventions.	Clinical validation, overcoming biases in AI models, ensuring transparency and explainability, and integrating tools seamlessly into healthcare systems remain hurdles.
[70]	Advanced just-in-time adaptive interventions can offer personalized, context-sensitive support for mental health, enhancing engagement and treatment outcomes by delivering the right intervention at the right time.	Algorithmic complexity, safeguarding data privacy, adapting to diverse user contexts and needs, and ensuring equitable access require ongoing research and development.
[71]	Digital health technologies for dementia care provide tools for symptom monitoring, cognitive support, and caregiver assistance, improving quality of life and potentially delaying institutionalization.	Ensuring usability for elderly populations, addressing technological acceptance and training needs, managing privacy and data protection, and ensuring affordability and accessibility pose challenges.
[72]	AI enables enhanced chronic disease health management by supporting risk prediction, treatment personalization, and efficient resource allocation, improving patient outcomes and system sustainability.	Integrating data from heterogeneous sources, clinical adoption barriers, ethical considerations, and ensuring equitable access across different populations are key concerns.
[73]	AI-driven language translation and interpretation in clinical settings can overcome communication barriers, improving patient-provider interactions, reducing errors, and enhancing care quality.	Ensuring translation accuracy, maintaining cultural sensitivity, securing patient data, and achieving provider and patient acceptance are important challenges.
[74]	Mobile health apps for inflammatory bowel disease empower patients with symptom tracking, medication reminders, and educational content, fostering self-management and improved outcomes.	Sustaining user engagement, ensuring data accuracy and reliability, integration with healthcare provider systems, and addressing privacy concerns are ongoing challenges.
[75]	Self-management support apps for spinal cord injury patients facilitate personalized rehabilitation, promote autonomy, and improve quality of life through tailored interventions and monitoring.	Adapting interventions to diverse functional levels, ensuring accessibility and usability, managing technological complexity, and maintaining patient motivation are key issues.
[76]	AI-based facial recognition technology offers a non-invasive, accessible screening tool for obstructive sleep apnea, potentially enabling early detection and intervention.	Validation of screening accuracy across populations, privacy and ethical concerns regarding biometric data, and integration into standard clinical pathways remain challenges.

**Table 6:** Description of the found mobile health apps declared as medical device.

[Ref.] First Author (Year; Country) *Title*	Medical Device Characteristics Database of Registration Device Name-Risk Class Code (Nomenclature Code(s) for EUDAMED; CND for Italian Database) Manufacturer (Country)–Link DESCRIPTION
[86] Veyron JH et al. (2024; France) *Postimplementation Evaluation in Assisted Living Facilities of an eHealth Medical Device Developed to Predict and Avoid Unplanned Hospitalizations: Pragmatic Trial*	Registered in EUDAMED **Presage Care**—Class I **Code:** 40192-General medicine diagnosis and monitoring instruments-medical device software **Manufacturer:** PRESAGE (FR)—https://presage.care/, accessed on 28 July 2025 **Market distribution:** Belgium (from 3 April 2023 to 30 November 2028); France (from 28 February 2020 to 30 November 2028) DESCRIPTION: Presage Care is a remote monitoring system for patients using electronic patient-reported outcome measures (data recorded by nurse assistants into a smartphone app) with a machine learning algorithm (resident to a secure server) to predict the risk of emergency hospitalizations with a prediction window of 7–14 days. The smartphone app belongs to the system, but the machine learning algorithm is not a built-in algorithm.
[87] Karakoyun et al. (2021; Germany) *Digital Medical Device Companion (MyIUS) for New Users of Intrauterine Systems: App Development Study*	Registered in EUDAMED **MyIUS**—Class IIa **Code:** V92-Medical device software-not included in other classes **Manufacturer:** BAYOOCARE GmbH [DE]—https://www.bayoocare.com/en/myius/, accessed on 28 July 2025 **Market distribution:** Belgium; Czechia; Denmark; Germany; Netherlands; Poland; Portugal; Sweden. DESCRIPTION: This device is a mobile phone–based medical app, that “uses an artificial intelligence–based bleeding pattern prediction algorithm to estimate a woman’s future bleeding pattern in terms of intensity and regularity” (citation from paper). This app supports the women after the placement of an intrauterine system, by recording menstrual bleeding information into a daily diary and predicting future bleeding patterns. From paper you can read, “The app is available free of charge in the App Store (Apple Inc) and Google Play Store (Google LLC)”.
[88] Atee et al. (2018; Australia) *Technical Note on the PainChek™ System: A Web Portal and Mobile Medical Device for Assessing Pain in People With Dementia*	Registered in the Italian Database **PAINCHEK APP**—Class I **Code:** Z12040282-General medicine therapeutic treatment instruments-software accessories **Manufacturer:** PAINCHEK LTD [AU]—https://www.painchek.com/, accessed on 28 July 2025 Representative (for non-European manufacturers): DONAWA LIFESCIENCE CONSULTING SRL [IT] **Market distribution:** Australia, United Kingdom, European Union (Italy from 17 October 2020), and other jurisdictions. DESCRIPTION: From the paper you can read, “*Using the smart-device camera to capture a short video of a person’s face, the App automatically identifies the face in real time, then maps the face to analyze facial expressions (using a built-in AI algorithm) indicative of the presence of pain*”.

**Table 7:** Description of the relevant studies dealing with regulatory issues regarding artificial intelligence.

Ref. First Author (Year; Country) *Title*	Regulation Issue	Description of the Study
[94] Shah SFH (2025; UK) *Ethical implications of artificial intelligence in skin cancer diagnostics: use-case analyses*	Efficacy and safety (*Robustness and safety*: ethical issues) and accountability (*accountability*: legal issue)	Analysis of the ethical issues in the use cases of two commercial mobile applications in the UK for artificial intelligence-assisted skin cancer diagnosis aimed at patients (SkinVision and Scanoma). The authors identified specific issues related to the development and use of each app. Of the two, only one app provided detailed information about how it works and how it was developed. However, for both apps, the issues identified related to the number, quality, and consistency of studies evaluating the effectiveness of the algorithm, as well as potential skin-tone biases that could lead to the exclusion of individuals with darker skin tones as target users. To make apps safer and more useful for all, the authors recommend more effective regulation to increase vendor accountability and ensure ethics by design through integration between developers, dermatologists, ethicists, and users. SkinVision (https://www.skinvision.com, accessed on 28 July 2025), available for download in Europe, Australia and New Zealand, is classified as a class I mark medical device in the European Union (EU) and Australia. Scanoma (https://www.scanoma.com, accessed on 28 July 2025), available for download in Australia, UK and US, is classified as a class I mark medical device in Australia.
[95] Späth J (2024; Germany, France, Spain) *Privacy-Preserving Federated Survival Support Vector Machines for Cross-Institutional Time-To-Event Analysis: Algorithm Development and Validation*	Data privacy (*Privacy and security*)	This study develops and validates a privacy-preserving federated survival support vector machine (SVM) designed for cross-institutional time-to-event analyses in medical research. The algorithm, implemented as a freely accessible app on the FeatureCloud platform, yields results very similar to centralized models, demonstrating high accuracy and robustness, even with site-dependent variations. This approach offers a valuable tool for collaborative research while respecting strict privacy regulations.
[96] Zawati MH (2024; Canada) *Does an App a Day Keep the Doctor Away? AI Symptom Checker Applications, Entrenched Bias, and Professional Responsibility*	Entrenched bias (*Fairness and inclusiveness*; ethical issue) and professional accountability (*Accountability*; legal issue)	This paper discusses the ethical and legal challenges of AI-powered symptom checker apps in mobile health, focusing on issues like bias from training data, demographic inequalities, and lack of regulation. Using examples like Babylon and Ada apps, it highlights concerns over entrenched biases and professional accountability, including uncertainties about safety reviews and liability. The authors emphasize the need for technical safeguards, neutral data training, and effective regulation to ensure these apps are safe, fair, and transparent, underscoring that addressing these issues is crucial for responsible development and deployment of AI in healthcare.
[97] Wongvibulsin S (2024; US) *Current State of Dermatology Mobile Applications with Artificial Intelligence Features*	Efficacy and safety (*Robustness and safety*), and transparency (*Transparency and explainability*)	An analysis of dermatology apps with artificial intelligence features available for download from the Apple and Android app stores was conducted. Forty-one apps were analyzed: 14 apps were based in the US, with only 2 providing a disclaimer for lacking US Food and Drug Administration approval; additionally, 14 apps were based in Europe, and only 2 possessed CE Mark (Conformité Européenne) status. Overall, the study found that the associated risks were a lack of consistent validation, transparency in algorithm development, user privacy and misleading user communication. To minimize risks, effective regulation is needed, including the establishment of standardized validation and evaluation criteria to ensure the efficacy, safety, and transparency of these applications.
[98] Alfano L (2024; Italy) *Psychotherapy, artificial intelligence and adolescents: ethical aspects*	Responsible implementation (*Accountability*), patient privacy (*Privacy and security*), and the human-AI interaction (*Robustness and safety*)	This article discusses the ethical considerations surrounding the use of AI in psychotherapy, emphasizing the need for careful regulation to ensure responsible implementation, patient safety and data privacy, and human-AI interaction.
[99] Ouellette S (2022; US) *Usefulness of Smartphones in Dermatology: A US-Based Review*	Regulation of health-related apps for diagnosing and treating diseases, such as medical devices (*All the basic principles*)	Review of the usefulness of mobile apps for dermatology, both for patients and providers. 15.7% of the apps analyzed used artificial intelligence. The article expresses concern about the lack of regulation of medical apps in the same way as medical devices, with the authors stating that the software function of apps used to diagnose and/or treat disease should be cleared by the FDA [79]. However, no skin cancer detection app appears to be regulated by the FDA and, unfortunately, is still freely available for download.
[100] Matin RN (2021; UK) *AI-based smartphone apps for risk assessment of skin cancer need more evaluation and better regulation*	Regulation of health-related apps for diagnosing and process of CE certification (*All the basic principles*)	The widespread adoption of artificial intelligence (AI)-based smartphone apps in healthcare must be supported by a strong scientific evidence base and properly regulated claims by app manufacturers. Unfortunately, current CE marking evaluation processes do not adequately protect the public from the risks of using these diagnostic apps, as many of these apps, when certified, turn out to be Class I medical devices, for which there is no need for independent regulatory review, as app developers “self-certify” and effectively apply the CE mark. In this scenario, the most “dangerous” apps are mainly those developed for dermatology, which, based on captured images of the skin and using machine learning techniques, give indications of suspicious lesions (skin cancer).
[101] Pashkov VM (2020; Ukraine) *Artificial Intelligence in Medical Practice: Regulative Issues and Perspectives*	Implementation of regulated AI in healthcare (*All the basic principles*)	This paper provides guidance for understanding the nature of AI in healthcare and the specifics of its regulation. The main issues related to the implementation of AI in healthcare stem from the nature of the technology itself and the complexity of ensuring legal support in terms of safety, efficacy, privacy, ethical issues and liability. The main challenge lies not in the technology, which is rapidly advancing and revealing new applications, but in the inadequacy of the existing legal framework. Indeed, one must ask whether the current legal framework is adequate to regulate AI in terms of safety, efficacy, and pre- and post-market monitoring, and to build a model of accountability in the context of AI use in healthcare.
[102] Martinez-Martin N (2018; US) *Ethical Issues for Direct-to-Consumer Digital Psychotherapy Apps: Addressing Accountability, Data Protection, and Consent*	Responsibilities and ethical obligations in artificial intelligence-led counseling and conversational agents in direct-to-consumer (DTC) digital psychotherapy (*All the basic principles*)	Direct-to-consumer (DTC) digital psychotherapy services that do not involve supervision by a mental health professional face many ethical challenges in protecting the person in therapy due to the lack of adequate regulation in this area. Indeed, there are no clear lines of accountability or associated ethical obligations to protect the user, his or her safety and privacy. This makes services that offer counseling guided by artificial intelligence and conversational agents even more problematic.

**Table 8:** Selected ISO/IEC applicable to this context.

Standard	Title/Scope	Relevance to AI-Powered Health Apps	Notes	Ref
ISO/TS 82304-2:2021	Health and wellness apps—Quality and reliability	Establishes requirements for health apps covering development, deployment, and lifecycle management	Useful for both consumer and clinically oriented apps, supports assessment and certification	[131]
IEC 82304-1:2016	Health software—General requirements for product safety	Provides general safety requirements for standalone health software, including AI components	Complements SaMD regulatory frameworks; applicable when software functions independently from hardware	[132]
ISO/IEC 42001:2023	AI Management Systems	Provides a framework for governance, transparency, risk management, and continuous improvement of AI systems	Supports safe and accountable deployment of AI in clinical contexts	[133]
ISO 13485:2016	Medical devices—Quality management systems	Quality management system requirements for manufacturers of medical devices	Required if the app is classified as a medical device (SaMD); ensures consistency in production and documentation	[134]
ISO 14971:2019	Medical devices—Risk management	Provides guidance for clinical risk assessment and mitigation	Critical for evaluating potential harms of AI-powered apps, including decision support features	[135]

**Table 9:** Selected applicable IT standards.

Standard	Title/Scope	Relevance to AI-Powered Health Apps	Notes	Ref.
ISO/IEC 27001:2022	Information security management systems	Establishes requirements for information security management, including risk assessment and mitigation	Essential for protecting patient data and AI algorithms in clinical settings	[136]
ISO/IEEE 11073-40101:2022	Health informatics—Device interoperability—Cybersecurity—Vulnerability assessment processes	Provides processes for secure interoperability between devices and IT systems	Particularly relevant for apps interacting with sensors, wearables, or medical devices	[137]
IEC/TR 80002-1:2009	Medical device software—Guidance on applying ISO 14971 to software	Guidance on applying risk management principles specifically to software	Supports safe deployment of AI-enabled apps within medical contexts	[138]

**Table 10:** Specific selected document.

Standard/Guideline	Title/Scope	Relevance to AI-Powered Health Apps	Notes	Ref.
AAMI TIR 34971:2023	Application of ISO 14971 to ML/AI	Provides guidance for risk management of AI/ML in medical devices	Directly relevant to AI-powered apps; ensures risk assessment covers adaptive algorithms	[139]
FG-AI4H DEL2.2:2022	Good practices for health AI applications	Development, validation, and compliance framework for AI in health	Complements formal standards with practical guidance for clinical and consumer apps	[140]

**Table 11:** Specific selected document.

Standard/Framework	Title/Scope	Relevance to AI-Powered Health Apps	Notes	Ref.
ISO/TS 82304-2:2021	Health and wellness apps—Quality and reliability	Provides a structured approach for app assessment and potential certification	Supports evaluation for clinical and consumer-facing apps	[131]
ISO/IEC 42001:2023	AI Management System	Facilitates continuous monitoring and governance of AI algorithms	Ensures responsible AI deployment and iterative improvement	[133]

## Data Availability

No new data was created.

## Supplementary Materials

The following supporting information can be downloaded at https://www.mdpi.com/article/10.3390/bioengineering13010054/s1, Section S1: Independent Quality Assessment of Included Studies; Table S1: mean scores assigned to ecah study (the study is anonymyzed); Table S2: The proposed search strings used for PubMed database saeraches in Section 3.2; Table S3: The proposed search strings for exploring the regulatory landscape surrounding AI-powered mobile health apps, in relation to both medical device and AI regulations in Section 4.
