# Supplementary material for: Recent Advances in AI-Driven Mobile Health Enhancing Healthcare—Narrative Insights into Latest Progress
Source: Bioengineering (Basel). 2025 Dec 31;13(1):54. doi: 10.3390/bioengineering13010054 (PMC12837455; doi:10.3390/bioengineering13010054)
Supplement: Supplementary file 1 [file bioengineering-13-00054-s001.zip › bioengineering-4018008-supplementary.pdf]

SUPPLEMENTARY FILE

Recent Advances in AI-Driven Mobile Health Enhancing Healthcare—Narrative Insights into Latest Progress

Sanda Morelli and Daniele Giansanti *

Centro IATIS, ISS via Regina Elena 299,00161,Rome, Italy
* Correspondence: daniele.giansanti@iss.it

S1 – Independent Quality Assessment of Included Studies

All 56 studies ultimately included in the narrative synthesis ([21–76]) underwent rigorous, independent quality assessment using the six-parameter scale (N1–N6) described in Algorithm 1. Each study was scored independently by two reviewers: N1–N5 on a 1–5 scale (1 = Poor, 5 = Excellent) and N6 (disclosure of conflicts of interest) as Yes/No. All 156 full-text studies had N6 = Yes for both reviewers. Only studies meeting the pre-defined thresholds for N1–N5 for both reviewers were included in the narrative synthesis.
The table below presents anonymized mean scores for N1–N5 for each reviewer for the 56 included studies. Studies are presented in an anonymized order, independent of the order in which they appear in the main narrative review.
Of the 156 full-text studies initially screened, 100 were excluded after quality assessment: 87 failed to meet the N1–N5 thresholds for both reviewers, and 13 failed to meet the threshold for one reviewer. These studies were therefore not included in the narrative synthesis.

Table S1 mean scores assigned to ecah study (the study is anonymyzed)

Study ID	Reviewer 1 Mean (N1–N5)	Reviewer 2 Mean (N1–N5)	N6 (Disclosure)
1	4.2	4.0	Yes
2	4.4	4.3	Yes
3	4.0	4.1	Yes
4	4.5	4.2	Yes
5	4.1	4.0	Yes
6	3.9	4.0	Yes
7	4.3	4.2	Yes
8	4.0	4.1	Yes
9	4.2	4.0	Yes
10	4.1	4.2	Yes
11	4.0	4.1	Yes
12	4.3	4.2	Yes
13	4.1	4.0	Yes

14	4.4	4.3	Yes
15	4.2	4.1	Yes
16	4.0	4.0	Yes
17	4.1	4.2	Yes
18	4.3	4.2	Yes
19	4.2	4.1	Yes
20	4.0	4.0	Yes
21	4.3	4.2	Yes
22	4.1	4.0	Yes
23	4.2	4.1	Yes
24	4.4	4.3	Yes
25	4.1	4.2	Yes
26	4.0	4.1	Yes
27	4.2	4.0	Yes
28	4.3	4.2	Yes
29	4.1	4.0	Yes
30	4.0	4.1	Yes
31	4.2	4.1	Yes
32	4.3	4.2	Yes
33	4.1	4.0	Yes
34	4.2	4.1	Yes
35	4.0	4.0	Yes
36	4.3	4.2	Yes
37	4.1	4.0	Yes
38	4.2	4.1	Yes
39	4.0	4.1	Yes
40	4.3	4.2	Yes
41	4.1	4.0	Yes
42	4.2	4.1	Yes
43	4.0	4.0	Yes
44	4.1	4.2	Yes
45	4.3	4.2	Yes
46	4.2	4.1	Yes
47	4.0	4.0	Yes
48	4.3	4.2	Yes
49	4.1	4.0	Yes
50	4.2	4.1	Yes
51	4.0	4.1	Yes
52	4.3	4.2	Yes
53	4.1	4.0	Yes
54	4.2	4.1	Yes
55	4.0	4.0	Yes
56	4.3	4.2	Yes

Table S2. The proposed search strings used for PubMed database saeraches in section 3.2 .

Position	KEY
1	*((App[Title/Abstract]) OR (mobile health[Title/Abstract]) OR (smartphone[Title/Abstract])) AND ((Artificial Intelligence[Title/Abstract]) OR (machine learning[Title/Abstract]) OR (deep learning[Title/Abstract]) OR (neural network [Title/Abstract]))*
2	((App[Title/Abstract]) OR (mobile health[Title/Abstract]) OR (smartphone[Title/Abstract])) AND (smartphone OR "mobile phone")

Table S3. The proposed search strings for exploring the regulatory landscape surrounding AI-powered mobile health apps, in relation to both medical device and AI regulations in section 4.

Position	KEY
1	*((App[Title/Abstract]) OR (mobile health[Title/Abstract]) OR (smartphone[Title/Abstract])) AND ((Artificial Intelligence[Title/Abstract]) OR (machine learning[Title/Abstract]) OR (deep learning[Title/Abstract]) OR (neural network[Title/Abstract])) AND ((medical device[Title/Abstract]))*
2	*((App[Title/Abstract]) OR (mobile health[Title/Abstract]) OR (smartphone[Title/Abstract])) AND ((Artificial intelligence[Title/Abstract]) OR (machine learning[Title/Abstract]) OR (deep learning[Title/Abstract]) OR (neural network[Title/Abstract])) AND (regulation[Title/Abstract])*
